# Enhancing medical image segmentation with HCE-Net: A heterogeneous feature collaborative extraction network

**DOI:** 10.1371/journal.pone.0359398

**Published:** 2026-11-03

**Authors:** Guoqing Zhu, Hongzhi Li, Han Cui, Zhanghao Ren, Jiaxi Wang

**Affiliations:** 1 School of Computer Science, Chengdu University, Chengdu, China; 2 Key Laboratory of Pattern Recognition and Intelligent Information Processing, Institutions of Higher Education of Sichuan Province, Chengdu University, Chengdu, China; 3 Key Laboratory of Digital Innovation of Tianfu Culture, Sichuan Provincial Department of Culture and Tourism, Chengdu University, Chengdu, China; University of Pisa, ITALY

## Abstract

Medical image segmentation occupies a pivotal role in clinical diagnosis and therapeutic planning. Conventional segmentation techniques struggle to accommodate the intricate representational attributes of medical images, while prevailing deep learning segmentation models predominantly adopt homogeneous single encoders and linear feature fusion paradigms, thereby exhibiting inherent limitations in contextual modeling, fine-grained feature excavation, and contour segmentation accuracy. To address such dilemmas, this paper proposes a Heterogeneous Collaborative Feature Extraction Network (HCE-Net). Establishing a dual-path heterogeneous encoding architecture, this network excavates complementary feature representations in a parallel manner. An Orthogonal Fusion Module (OFM) is elaborately devised to integrate Sobel edge priors with multi-scale convolutions, enabling the precise capture of edge and contour characteristics. A Diversified Receptive Field Module (DFM) is constructed to consolidate multi-scale detail manifestations and preserve global contextual cues. Furthermore, Adaptive Aggregation Connection (AAC) is embedded within skip connection branches to supplant conventional simplistic concatenation, efficaciously mitigating the technical bottleneck of heterogeneous feature fusion. Comparative experiments conducted on three publicly accessible medical datasets, encompassing dental radiographs, pulmonary CT scans, and retinal vascular images, demonstrate that HCE-Net substantially elevates segmentation accuracy, enhances boundary integrity, and ameliorates the general robustness of the model. It manifests distinguished application prospects across diverse medical image segmentation scenarios.

## Introduction

Medical image segmentation aims to partition medical images into multiple subregions with homogeneous internal properties and distinct inter-regional discrepancies, so as to accurately extract regions of interest. It furnishes crucial support for precise disease diagnosis, surgical planning, lesion localization, and quantitative analysis of tissues and organs. The precision of segmentation results directly governs the reliability of subsequent clinical procedures and decision-making [1]. For instance, accurate lesion segmentation enables clinicians to intuitively acquire the size, morphology and spatial distribution of lesions, thereby formulating more efficacious therapeutic strategies. Accordingly, improving the segmentation accuracy of medical images bears profound practical significance.

Medical image segmentation has evolved from traditional automatic segmentation techniques. Classical approaches primarily encompass edge-based, threshold-based, region-based and morphology-driven segmentation algorithms [2]. Such methods boast lucid principles and favorable interpretability, yet they heavily hinge on manually crafted features. Consequently, they struggle to adapt to complex medical images characterized by blurred boundaries, low contrast and substantial individual differences, failing to meet the stringent high-precision requirements in clinical practice.

The rapid advancement of deep learning has spearheaded transformative breakthroughs in medical image segmentation. Since the proposition of U-Net [3] in 2015, the encoder–decoder architecture has evolved into the predominant paradigm for medical image segmentation. On this basis, numerous optimized variants have emerged progressively. U-Net++ [4] adopts a nested architectural design to strengthen multi-level feature fusion; TernausNet [5] integrates a pre-trained VGG encoder [6] to enhance feature representation capability; V-Net [7] leverages volumetric convolution to accomplish three-dimensional medical image segmentation while preserving intricate spatial structural information; SegNet [8] optimizes memory utilization through refined encoder–decoder design. Furthermore, a series of advanced variants including CNS-UNet [9], MLFA-UNet [10], CSWin-UNet [11] and DSML-UNet [12] further boost segmentation performance by incorporating multi-scale representations, attention mechanisms and large-kernel convolutions.In recent years, Transformer [13] architectures have been extensively deployed in segmentation tasks owing to their extraordinary capacity for global contextual modeling, with representative works such as TransUNet [14] and SwinUNet [15]. Emerging research further integrates convolutional neural networks (CNN) with Transformer modules to construct dual-encoder or hybrid architectures, exemplified by DMSA-UNet [16], HmsU-Net [17] and NHBS-Net [18]. These hybrid designs harmonize fine-grained local detail perception and long-range global dependency modeling, achieving comprehensive performance enhancement.

Despite their inherent merits, convolutional neural networks are constrained by intrinsic limitations in capturing long-range dependencies. As network depth increases, shallow low-level cues such as edge contours, tonal attributes and positional information are prone to be neglected during model optimization. A single encoder struggles to balance fine local detail perception and comprehensive global contextual reasoning. Continuous downsampling operations inevitably induce the loss of edge details and subtle semantic features, ultimately resulting in inaccurate contour delineation. Existing dual-encoder architectures fail to achieve functional complementarity, while most skip connections rely on simplistic concatenation operations. Such designs hinder the effective integration of heterogeneous feature representations and incur redundant computational overhead simultaneously.

To address the aforementioned challenges, this paper proposes a heterogeneous collaborative feature extraction image segmentation network based on a dual-path feature extraction stream. Specifically, guided by the principles of high efficiency and simplicity, the network architecture incorporates an efficient attention mechanism that integrates the advantages of edge detection while preserving the detailed features of images, constructing a heterogeneous dual-feature stream for image feature extraction. The specific contributions of this paper are as follows:

(1) A dual-path heterogeneous collaborative feature extraction network, HCE-Net, is proposed to address the dilemmas that a single encoder struggles to capture the contextual detail information and edge feature information of images, as well as the difficulty in fusing features caused by linear fusion.
(2) An Orthogonal Fusion Module (OFM) is designed to integrate Sobel edge priors with multi-scale convolutions for extracting precise edge features of images, and a Diversified Receptive Field Module (DFM) is devised to preserve global detail features through multi-scale convolutions and channel attention.
(3) An Adaptive Aggregation Connection (AAC) is designed to achieve adaptive fusion of heterogeneous features, aiming to solve the problem that linear fusion methods are difficult to fuse features from different feature streams.

## Related work

This section briefly reviews the advancements of deep learning and encoder architectures in the field of medical image segmentation, and concisely introduces the applications of edge detection operators and attention mechanisms within image segmentation tasks.

### Advancements of deep learning and encoder architectures in image segmentation

Medical image segmentation not only assumes an indispensable role in clinical diagnosis but also serves as the cornerstone for other medical image processing tasks. With the advent of the computer age, deep learning-based image segmentation techniques have evolved into an irreplaceable tool in the realm of medical image processing. The Fully Convolutional Network (FCN) [19] stands as one of the earliest applications of deep learning in medical image segmentation. By substituting fully connected layers with convolutional layers, it accomplishes pixel-level segmentation of images of arbitrary dimensions. Nevertheless, FCN suffers from inadequacies in detail handling, leaving ample room for enhancement in segmentation accuracy. U-Net is a network architecture meticulously designed for biomedical image segmentation, comprising two integral components: an encoder and a decoder. The encoder undertakes the task of feature extraction, while the decoder maps these extracted features back to the original image space, facilitating refined and precise segmentation. U-Net and its enhanced variants (e.g., U-Net++, U-Net3+ [20]) have exhibited remarkable performance in medical image segmentation tasks, particularly when confronted with images characterized by intricate structures and delicate details.

Residual networks tackle the gradient vanishing issue in deep networks by introducing residual connections, thereby augmenting the training efficiency and overall performance of the network. The application of ResNet [21] in medical image segmentation contributes significantly to elevating the accuracy and stability of the model. With the proliferation of Transformers in the field of image processing, their self-attention mechanism, which targets global image patches, can effectively compensate for the deficiency of global information in Convolutional Neural Networks (CNNs). Subsequently, a diverse array of Transformer-based variants has emerged. For instance, TransUNet integrates the Transformer with U-Net, whereas VT-UNet [22] is a U-Net architecture constructed on the foundation of the Transformer. Certain networks combine CNNs and Transformers to form dual-encoder structures: I2U-Net [23] adopts a dual-path U-Net structure; FFUNet [24] replaces conventional skip connections with learnable feature fusion modules; TMSDNet [25] employs Transformers for single-view and multi-view 3D reconstruction, aiming to rectify the oversight of the pivotal role played by the deep multi-scale representation of objects in the voxel feature domain. However, FFUNet relies on a single encoder; the dual-path symmetric structure of I2U-Net fails to achieve functional complementarity; the CNN+Transformer parallel architecture incurs greater overhead and computational load after incorporating Transformer modules. Consequently, there remains substantial room for improvement in the performance and other aspects of the dual-path feature extraction architecture.

### Edge operators and attention mechanisms

Edge detection operators play an indispensable role in image segmentation. They are primarily employed to identify regions in images where pixel values undergo significant variations, namely edges, thereby facilitating image segmentation. The fundamental principle of edge detection operators lies in recognizing edges by calculating changes in the grayscale values of pixels within the image. In images, edges typically manifest as step-like or roof-shaped variations in grayscale values. To detect these variations, edge detection operators traverse each pixel in the image and compute the grayscale differences between the pixel and its neighboring pixels. Based on the magnitude and direction of these differences, the operator can determine whether the pixel lies on an edge. Common edge detection operators include the Roberts operator [26], Canny operator [27], and Prewitt operator [28]. Among them, the Sobel operator [29] incorporates operations similar to local averaging, thus exerting a smoothing effect on noise and effectively eliminating its impact. The Sobel operator applies weighting to the influence of pixel positions, and due to its simplicity, ease of implementation, and noise-smoothing capability, it has been widely utilized in image segmentation. AE-Unet [30] integrates the Sobel operator into the first two layers of skip connections, aiming to enhance the ability to extract edge features. The attention mechanism originates from research on human vision. To utilize limited visual information processing resources, humans selectively focus on critical regions while ignoring irrelevant information. Over the past few years, various typical attention mechanisms have emerged in medical image segmentation research, such as SE [31], CBAM [32], and ECA [33]. These mechanisms suppress irrelevant regional parts in the input image and highlight the features of local regions of interest. Channel attention mechanisms focus on the information between image channels; by learning the relationships among different channels, they enhance the expression of feature channels relevant to specific tasks. Spatial attention mechanisms concentrate on the spatial dimension of images; by capturing the correlation between different pixels and utilizing weight distribution, they strengthen the feature expression of regions closely related to the segmentation task. Ni et al. added an Enhanced Attention Module (AAM) to RAUNet to fuse multi-level features and capture contextual information, helping to address the specular reflection problem [34]. MA-UNet [35] resolves the issue of feature misalignment by integrating multi-scale features and a mixed attention mechanism. Fan et al. introduced an Attention Fusion Module (AFM) to fuse the output features of CNN and Transformer branches, enabling the extraction and utilization of spatial and channel information in images [36]. Single-scale feature extraction struggles to cope with the complex and variable structures and textures in medical images. The emergence of multi-scale attention mechanisms allows models to capture image features at different scales, thereby better adapting to the characteristics of medical images. Multi-scale attention mechanisms are not confined to medical image segmentation of a single modality or a single disease; instead, they are gradually expanding to applications across modalities (such as CT and MRI) and across diseases (such as tumors and organs). This trend contributes to the construction of a more versatile and flexible medical image segmentation framework. However, there remains a paucity of research that unifies traditional edge priors, multi-receptive field convolutions, and adaptive attention fusion into a cohesive segmentation framework.

## Methodology

This section elaborates on the basic implementation procedures, fundamental modules, and operational processes of our proposed model (HCE-Net).

### Overall network architecture

The architecture of the heterogeneous collaborative feature extraction image segmentation network (HCE-Net) established in this study is illustrated in Fig 1. HCE-Net adopts a heterogeneous encoding path. It is worth noting that, unlike the single encoding of U-Net and the CNN-Transformer hybrid encoding of TransUNet and SwinUNet, HCE-Net employs a functionally decoupled heterogeneous dual-encoding stream, with one dedicated to edges and the other to details. The HCE-Net structure utilizes two parallel encoders with different structures to extract image features: the edge contour path embeds an Orthogonal Fusion Module (OFM) to focus on extracting contour and boundary information, while the global detail path embeds a Diversified Receptive Field Module (DFM) to capture abundant texture and contextual details. In addition, we adopt Adaptive Aggregation Connection (AAC) for skip connections, which leverages second-order local-global attention to dynamically weight the outputs of the two paths, thereby addressing the information imbalance caused by linear fusion. The fused features are then concatenated with the features upsampled by transposed convolution from the bottleneck layer, gradually restoring image details.

**Fig 1:**
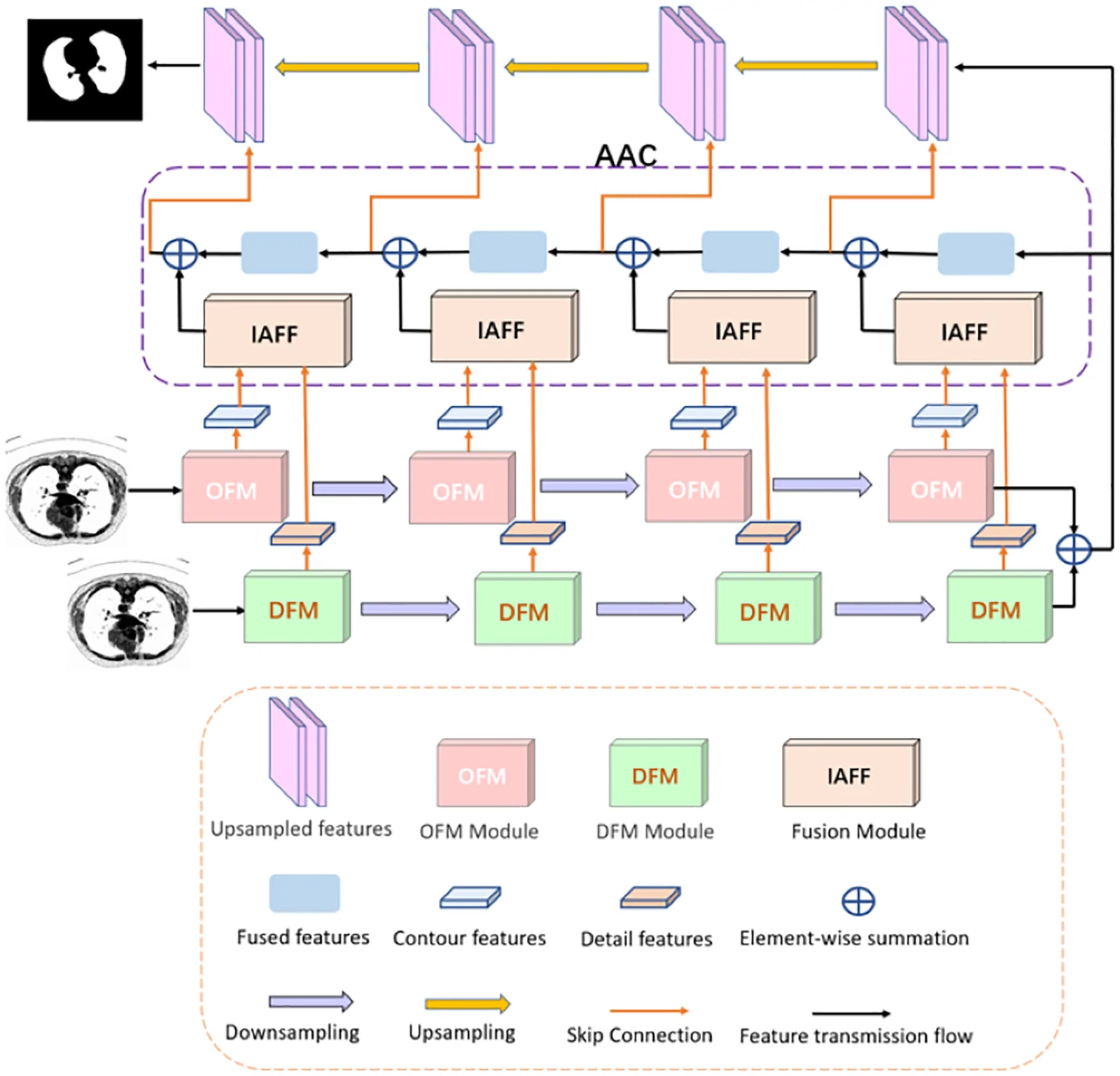
The Architectural Diagram of HCE-Net.

### Orthogonal fusion module

The structure of the Orthogonal Fusion Module (OFM) is illustrated in Fig 2. Distinct from existing methods, OFM innovatively integrates the non-learnable Sobel operator with a trainable deep convolutional network, infusing explicit edge priors into the network at an extremely low computational cost. Its core process is divided into three stages: First, bidirectional gradient perception: the Sobel operator is employed to calculate gradient maps in the horizontal and vertical directions respectively, edge intensity maps are obtained through square root operation, and enhanced by ReLU. This step introduces no learnable parameters, endowing the network with physically interpretable edge perception capability from the first layer—a inherent advantage of pure CNN designs over Transformer-based models in boundary localization. Second, multi-scale feature learning: the gradient-enhanced features are input into 3 × 3 and 5 × 5 convolution branches in parallel. Compared with single-scale or large-kernel convolutions, this dual-branch design can simultaneously capture fine edges (3 × 3) and wider contour transition zones (5 × 5) without significantly increasing parameters. Experimental verification shows that the combination of 3 × 3 + 5 × 5 achieves the optimal balance between edge segmentation accuracy and computational load. Finally, gradient-preserving connection and feature refinement: prior to fusion, we concatenate the initial edge features with the outputs of the dual-branch convolutions, restructure channels through 1 × 1 convolution, and add a global residual connection. This design ensures that even if deep convolutions weaken edge responses, the original gradient information can still directly reach subsequent layers, effectively alleviating the attenuation of edge information in deep networks. Eventually, a 3 × 3 convolution layer refines the fused features to enhance the consistency of edge responses. In summary, OFM not only retains the accuracy and interpretability of traditional edge detection operators but also leverages the advantages of deep learning in multi-scale feature extraction. Moreover, the entire module consists solely of convolutions and the Sobel operator, without the need for self-attention mechanisms, providing crucial support for HCE-Net to achieve precise contour segmentation while maintaining high efficiency. The specific operations of the Sobel operator are presented in Table 1.

**Table 1:** Implementation process for Sobel operators.

Algorithm 1: Sobel Edge Detection using Convolutional Filters
1: Input Feature Map
2: Defining the Sobel Filter
(1) A filter utilized for detecting horizontal edges sobel_x: [[−1, 0, 1],
[−2, 0, 2],
[−1, 0, 1]]
(2) A filter employed for detecting vertical edges sobel_y: [[−1, −2, −1],
[0, 0, 0],
[1, 2, 1]]
3: Convert the sobel_x and sobel_y into tensor formats.
4: Apply the Sobel filter for convolution.
5: Compute the gradient magnitude.
7: Apply batch normalization and the ReLU activation function.
8: Return the feature map processed through Sobel edge detection, batch normalization, and ReLU activation function.
9: Output the processed feature map after applying Sobel edge detection.

**Fig 2:**
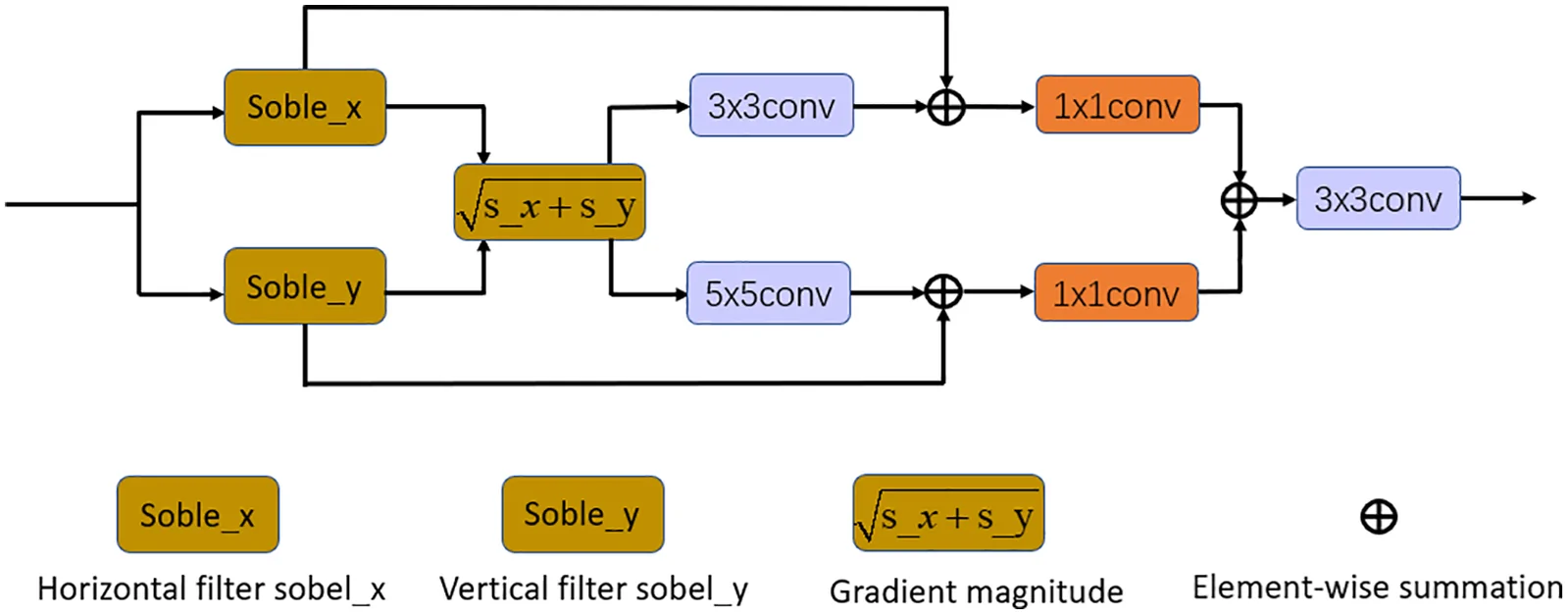
Diagram of the Orthogonal Fusion Module.

### Diverse field module

The Diversified Receptive Field Module (DFM), illustrated in Fig 3, is specifically designed in this study for the extraction of global details and texture features, serving as the core unit of the detail-aware path in the heterogeneous dual-encoding stream of HCE-Net. Distinct from traditional multi-scale convolutions or simplistic attention structures, DFM innovatively integrates channel attention stacking, cross residual connections, and multi-scale convolutions in an organic manner. It achieves efficient capture of fine-grained textures, local structures, and global context without significantly increasing computational load, addressing the limitations of single convolutions with limited receptive fields and the easy loss of detail information. Specifically, DFM first constitutes a Feature Calibration Unit (FCU) through three consecutive channel attentions and residual connections, dynamically weighting and optimizing feature channels to enhance responses to key details in medical images. Subsequently, the FCU is cross-series connected with 3 × 3, 5 × 5, and 7 × 7 multi-scale convolutions combined with residual connections to construct an adaptive multi-receptive field structure, enabling the model to simultaneously capture detail and contextual information at different scales. This cross-type structure avoids redundant computation of traditional parallel multi-branches, maintaining lightweight properties while expanding the coverage of receptive fields. Among them, the channel attention extracts global information through dual branches of global average pooling and maximum pooling, learns channel weights via shared linear layers, and ultimately completes feature recalibration using the Sigmoid function and element-wise multiplication, allowing the network to focus more on effective detail features. Incorporating DFM as the core convolution unit of the detail extraction branch in the heterogeneous encoding stream can significantly alleviate texture loss caused by ordinary convolutions during downsampling. It forms a complementary heterogeneous feature stream—one for edges and one for details—together with the Orthogonal Fusion Module (OFM), providing crucial support for HCE-Net to achieve high-precision segmentation.

**Fig 3:**
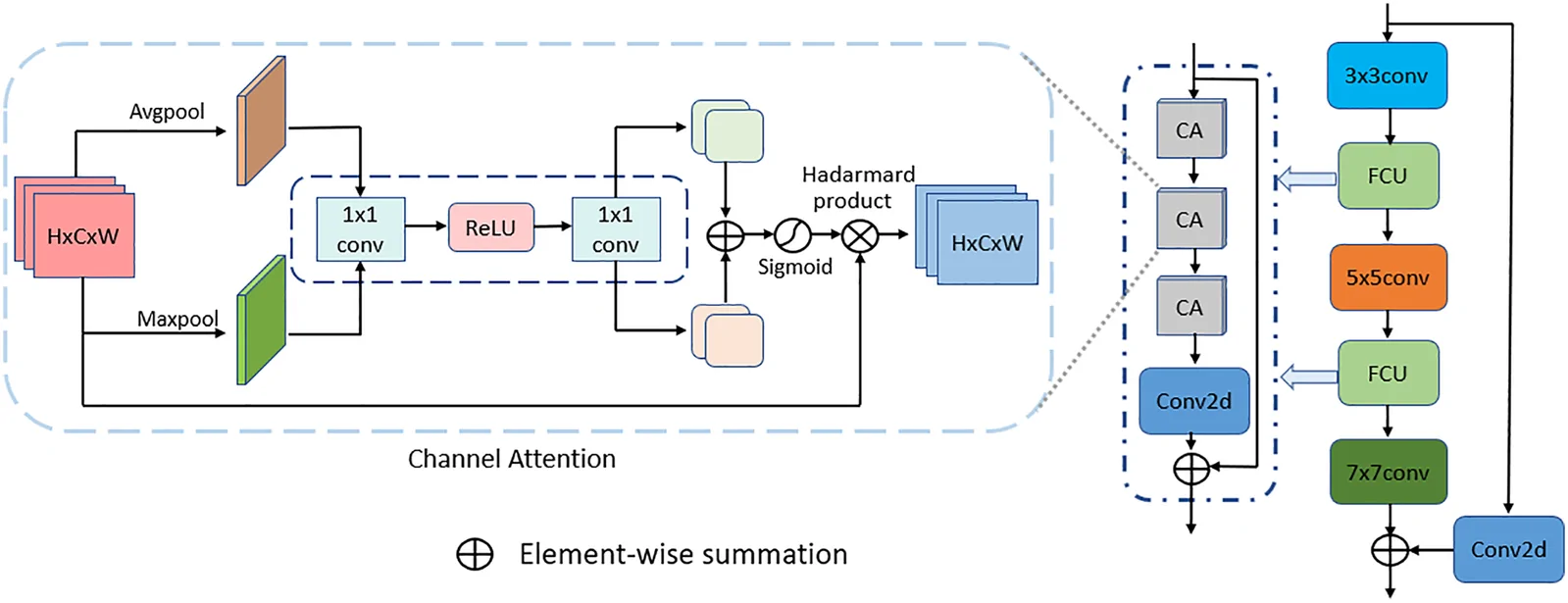
Diagram of the Diverse Field Module.

### Adaptive aggregation connection

The structure of the Adaptive Aggregation Connection (AAC) is illustrated in Fig 4. To address the problem that heterogeneous collaborative coding path features are difficult to directly fuse through traditional skip connections due to differences in scale, semantics, and distribution, this paper redesigns the adaptive aggregation connection AAC suitable for dual paths based on IAFF [37]. AAC is a layer-by-layer progressive feature fusion module adapted to the HCE-Net architecture, which is embedded in the branch of each skip connection. Specifically, before the features of the corresponding layers of the two paths enter the decoder, they first undergo in-depth fusion through AAC, enabling the contour features from the edge path (OFM) and the texture features from the global detail path (DFM) to complement each other at the earliest moment of contacting the decoder, thus avoiding semantic aliasing caused by simple concatenation in the decoder. Taking the two heterogeneous feature paths of HCE-Net as input, this module performs adaptive weighting and iterative fusion on the edge feature stream and the detail feature stream, effectively overcoming the problems of feature conflict and information loss caused by traditional concatenation or simple weighting. The features fused by AAC are then combined with the upsampled features of the decoder, which can accurately compensate for the detailed information and contour features lost during the downsampling process. Unlike directly using IAFF or traditional skip connections, the AAC proposed in this paper is specially customized for the heterogeneous dual-path structure of HCE-Net. By working in synergy with the OFM and DFM modules, it achieves efficient alignment and complementary enhancement of heterogeneous features. While improving the fusion quality, it significantly enhances the ability of skip connections to retain edge and detail information, ultimately improving the segmentation accuracy.

**Fig 4:**
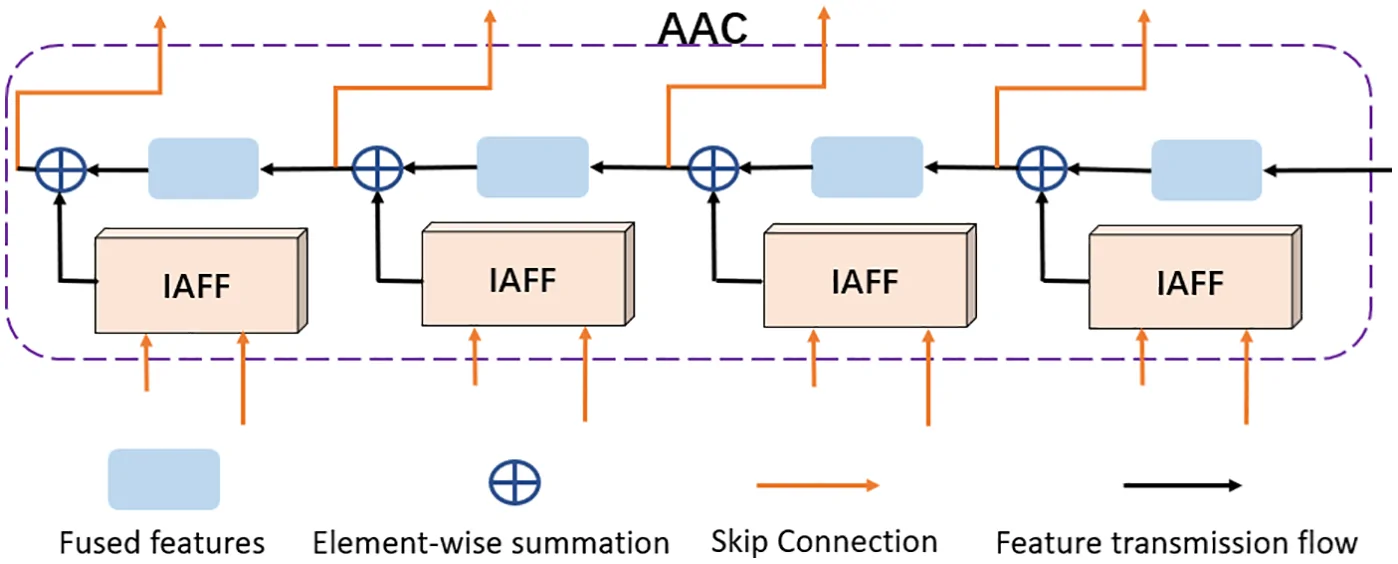
Diagram of Adaptive Aggregation Connection.

The structure of the Iterative Attention Feature Fusion (IAFF) mechanism is illustrated in Fig 5. This mechanism achieves the complementarity and enhancement of multi-source information by fusing feature maps from different paths, thereby obtaining more robust feature representations. IAFF integrates both local attention and global attention simultaneously, which can focus on the fine-grained structure and global context of feature maps respectively, improving the accuracy and efficiency of feature fusion. In this paper, we directly adopt the existing IAFF module as the basic fusion unit, and perform customized adaptation and reconstruction for the dual heterogeneous path collaborative structure and skip connection scenario of HCE-Net, ultimately forming the Adaptive Aggregation Connection (AAC) proposed in this paper. The core idea of IAFF is to adaptively weight channel features in the multi-scale dimension and maintain the lightweight nature of the module through 1 × 1 pointwise convolution. It consists of two branches: the local feature branch 𝐿(𝑋): directly performing channel attention learning on the feature map; the global feature branch 𝐺(𝑋): first extracting global information through global average pooling, and then performing channel attention learning. The calculation formula of local channel attention $L\left(x\right)\in R^{C\times H\times W}$ is:

**Fig 5:**
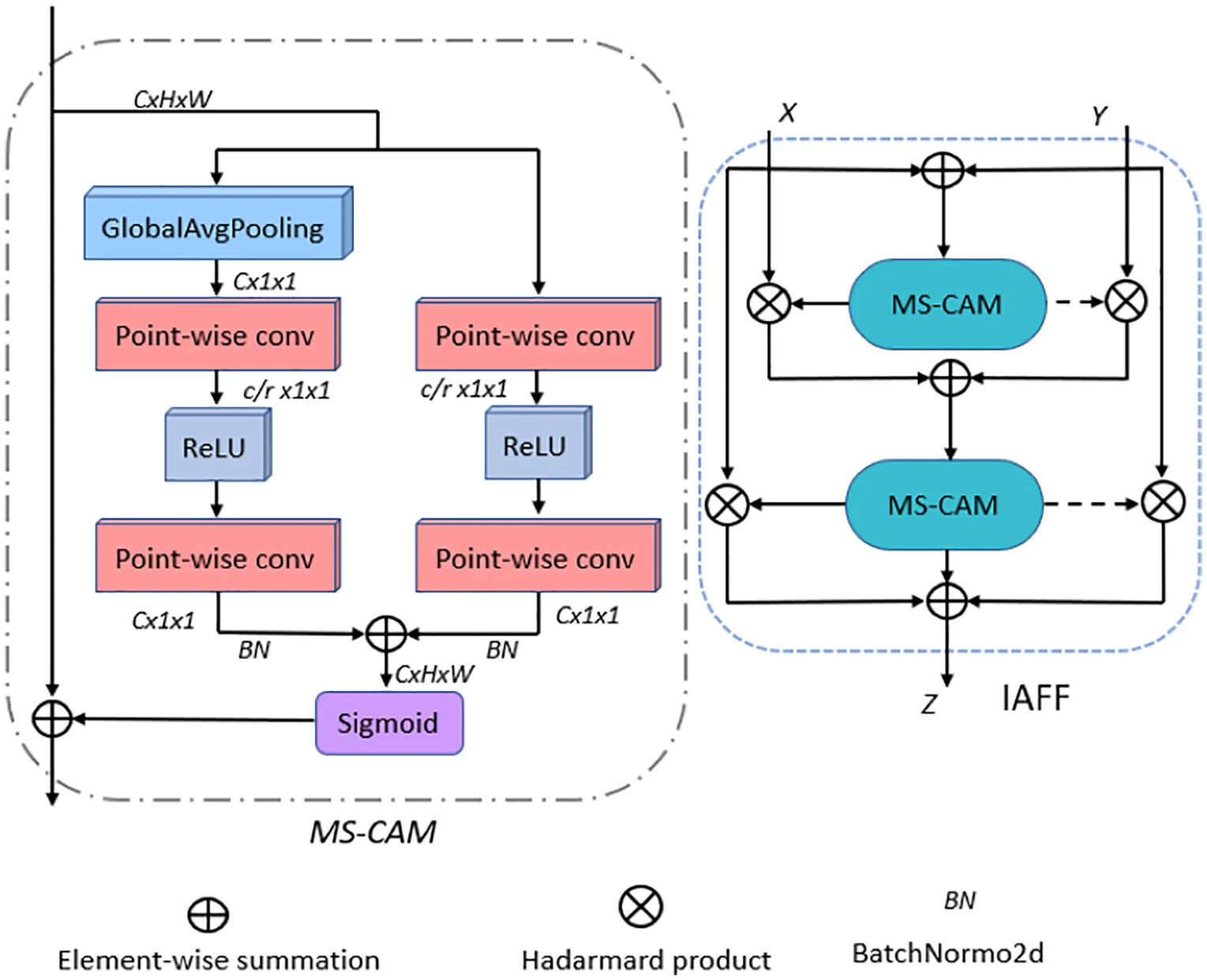
Diagram of the Iterative Attention Feature Fusion Mechanism.


$$\begin{array}{c} L\left(x\right)=B\left\{PWconv2\left(\delta \left(B\left(PWconv1\left(x\right)\right)\right)\right)\right\} \end{array}$$ (1)

Among them, *PWConv*_*1*_and *PWConv*_*2*_are 1 × 1 convolutions, which complete channel dimension reduction and dimension elevation respectively, and 𝛿 is the ReLU activation function. The global channel attention 𝐺(𝑋) adds a global average pooling operation on the basis of 𝐿(𝑋). The final attention output 𝑋′ is:


$$\begin{array}{c} X^{\prime }=X⊗M\left(X\right)=X⊗\sigma \left(L\left(x)⊕G(X\right)\right) \end{array}$$ (2)

Among them, ⊕denotes broadcast addition, ⊗denotes element-wise multiplication, and 𝜎 is the Sigmoid activation function. In the long skip connection scenario of this paper, we extend the input as follows: $X\in R^{C\times H\times W}$: detail feature map from the DFM module; $Y\in R^{C\times H\times W}$: contour feature map from the OFM module. The fusion process can be expressed as:


$$\begin{array}{c} Z=M(X⊎Y)⊗X+(1-M(X⊎Y))⊗Y \end{array}$$ (3)

The notation ⊎ signifies primordial feature fusion, wherein correlative weights are self-optimized through adaptive network learning and bounded within the interval [0,1]. Departing from the direct deployment of primitive IAFF, this study refashions its fundamental mechanism and embeds the refined paradigm into the AAC module. Such bespoke architecture adeptly reconciles discrepancies in spatial dimension, semantic connotation and statistical distribution between dual heterogeneous feature streams, thereby overcoming the intractable dilemma of cross-path feature amalgamation. Empirical investigations substantiate that the IAFF-augmented AAC module markedly elevates the integration proficiency of heterogeneous representations, conferring upon HCE-Net conspicuous advancements in structural boundary maintenance and intricate detail reconstruction. As delineated in Fig 5, 𝑋 characterizes the texture-enriched feature map, 𝑌 embodies the contour-oriented feature map, and $Z\in R^{C\times H\times W}$ manifests the consolidated feature embedding derived from AAC.

## Experiments

To demonstrate the superior performance of the proposed network architecture, we conducted comparative experiments with several classic and advanced networks. Additionally, corresponding ablation experiments were designed. Furthermore, this subsection will detail the specific experimental aspects of this study. The source codes and model weights are publicly accessible at:

https://github.com/qiangxuange/HCE-net/

### Datasets

#### Pulmonary CT lesions (PCL).

This data set is provided by K Scott Mader on the Kaggle website, we obtained two-dimensional images, including 267 CT images and ground truth, with a pixel size of 512 × 512. The ataset can be accessed via the following link: https://www.kaggle.com/datasets/kmader/finding-lungs-in-ct-data/data.

#### The MICCAi-tooth-segmentation dataset comprises 1998 images(tooth).

Each sized 640 × 320 pixels, designated for the task of tooth segmentation on provided two-dimensional panoramic X-ray image data. These images often exhibit noise, leading to poor quality or even blurriness. Additionally, some tissues surrounding the teeth bear high resemblance to the teeth themselves, increasing the segmentation difficulty. The ataset can be accessed via the following link: https://tianchi.aliyun.com/dataset/156596

#### Digital retinal images for vessel extraction (DRIVE).

It is specifically dedicated to retinal vessel segmentation. It holds significant importance in the field of medical image processing, particularly for the study of retinal pathologies such as screening and diagnosis of diabetic retinopathy (DR). This dataset can be accessed at https://drive.grand-challenge.org/.

The minimal data set, original data for tables and figures, and code supporting the results of this study are available at: https://github.com/qiangxuange/HCE-net/.

All data are freely accessible without restriction.

### Experimental details

This section elaborates upon the experimental settings and evaluation metrics adopted throughout this research.

#### Experimental environment and settings.

All experiments are implemented upon the PyTorch deep learning framework, with Python 3.10 and CUDA 11.8 deployed on a single NVIDIA graphics card equipped with 24 GB video memory. Regarding dataset partitioning and validation strategies, five-fold cross-validation is conducted across all datasets to guarantee the credibility and persuasiveness of experimental outcomes. The complete dataset is randomly divided into five equal subsets. In each iteration, one subset serves as the test set while the remaining four are designated for training. After five cyclic trials, the final results are presented as the mean value plus or minus standard deviation, effectively eliminating biases induced by data partitioning. All medical images are uniformly resized to a resolution of 256 × 256 pixels and normalized with a mean of 0.5 and a standard deviation of 0.5. During the training phase, random horizontal flipping and arbitrary rotation are introduced as data augmentation tactics to strengthen model generalization and mitigate overfitting risks. The Adam optimizer is adopted with an initial learning rate of 0.0001, alongside a cosine annealing scheduler for adaptive learning rate adjustment. The batch size is set to 4, and the maximum training epoch is limited to 100. A composite loss function combining Dice loss and cross-entropy loss is utilized for model optimization. When operating on the 24 GB NVIDIA graphics card with an input size of 256 × 256, the inference latency of HCE-Net for a single image reaches approximately 12 milliseconds, which sufficiently meets the efficiency requirements of clinical auxiliary diagnosis.

### Evaluation metrics

By constructing a confusion matrix, we employed four fundamental criteria: TP represents the number of instances where the model predicts a positive sample and the true value is also positive. TN represents the number of instances where the model predicts a negative sample and the true value is also negative. FP represents the number of instances where the model predicts a positive sample but it is actually negative. FN represents the number of instances where the model predicts a negative sample but it is actually positive. Based on these four basic classifications, the following metrics are introduced:


$$\begin{array}{c} Acc=\;\frac{\sum TP\;+\;\sum TN}{\sum TP\;+\;\sum TN\;+\;\sum FP\;+\;\sum FN}\times \text{100}\% \end{array}$$ (4)

$$\begin{array}{c} Dice=\;\frac{2\;\sum TP}{\sum FP\;+\;2\;\sum TP\;+\;\sum FN}\times \text{100}\% \end{array}$$ (5)

$$\begin{array}{c} Jaccard=\;\frac{\sum TP}{\sum FP\;+\;\sum TP\;+\;\sum FN}\times \text{100}\% \end{array}$$ (6)

$$\begin{array}{c} \;Precision=\;\frac{\sum TP}{\sum TP\;+\;\sum FP}\times \text{100}\% \end{array}$$ (7)

$$\begin{array}{c} Recall=\;\frac{\sum TP}{\sum TP\;+\;\sum FN}\times \text{100}\% \end{array}$$ (8)

$$\begin{array}{c} Specificity=\;\frac{\sum TN}{\sum TN\;+\;\sum FP}\times \text{100}\% \end{array}$$ (9)

Among them, Acc represents the proportion of pixels correctly predicted by the model, including true positives and true negatives. Dice measures the similarity between predictions and true data, calculated by dividing twice the intersection by the sum. Jaccard, also known as Intersection over Union (IoU), is the ratio of the intersection between predictions and true data to their union. Precision represents the proportion of pixels predicted as positive that are actually positive. Recall represents the proportion of all pixels that are actually positive and are correctly predicted as positive. Specificity represents the proportion of all pixels that are actually negative and are correctly predicted as negative.

### Results and visualization

To validate the effectiveness of the proposed network and its various modules in this study, this section will present the visual results of the experiments and the outcomes of ablation studies.

#### Experimental analysis.

All experimental outcomes are reported as the mean ± standard deviation derived from five-fold cross-validation, where the standard deviation is calculated from five independent trials to reflect the stability of model performance. To verify the statistical reliability of performance improvements, a t-test is conducted for comparative analysis between HCE-Net and the baseline U-Net. The obtained results reveal a p-value below 0.05, demonstrating that the performance gains possess distinct statistical significance.

Under identical experimental configurations, comprehensive comparisons are conducted against eleven prevailing medical image segmentation models, encompassing canonical CNN architectures including U-Net [3], SegNet [8], ResUnet [21], AMSUnet [38], Channel-Unet [39], Attention-Unet [40], and AAU-net [41], as well as state-of-the-art CNN‑Transformer hybrid paradigms such as SwinUnet [15], TransUnet [14], UNETR [42], and UTnet [43]. Quantitative results of all competing models across the TOOTH, PCL and DRIVE datasets are summarized in Table 2. Numerical observations evidently demonstrate that the proposed HCE-Net achieves superior performance in core segmentation metrics over all three datasets and exhibits compelling advantages against contemporary CNN‑Transformer hybrid frameworks. On the TOOTH dataset with intricate contour segmentation challenges, the Jaccard index of HCE-Net surpasses TransUnet by 4.291%, SwinUnet by 25.309%, UNETR by 9.141%, and UTnet by 12.480%. For the pulmonary CT-based PCL dataset, the Jaccard coefficient increases by 1.949%, 3.361%, 1.335% and 0.958% in comparison with TransUnet, SwinUnet, UNETR and UTnet, respectively. Regarding the DRIVE dataset characterized by sophisticated edge detail extraction for retinal vessels, HCE-Net attains Jaccard improvements of 13.484%, 36.067%, 2.678% and 11.696% over TransUnet, SwinUnet, UNETR and UTnet correspondingly. Furthermore, HCE-Net yields optimal values in critical metrics including Acc, Dice and Specificity across all evaluated datasets. Compared with the baseline U-Net, its accuracy is elevated by 1.189%, 0.185% and 2.168% on the TOOTH, PCL and DRIVE datasets respectively. Such consistent validations substantiate the high congruence between segmented predictions and ground-truth annotations. The presented model manifests remarkable capacity in capturing target morphological contours, delineating precise boundary information and excavating fine-grained contextual features, while maintaining robust generalization across diverse medical imaging scenarios.

**Table 2:** The indicator results of HCE-Net and different comparison networks on three datasets.

Datasets	Networks	Acc(%)	Dice(%)	Jaccard(%)	Pre(%)	Recall(%)	Spe(%)
**TOOTH**	U-Net	88.03 ± 0.16	73.73 ± 0.22	58.87 ± 0.28	60.41 ± 0.34	96.29 ± 0.21	87.43 ± 0.13
	SegNet	87.91 ± 0.15	74.31 ± 0.23	59.32 ± 0.29	60.01 ± 0.32	98.49 ± 0.19	86.85 ± 0.14
	ResUnet	88.85 ± 0.14	75.90 ± 0.21	59.33 ± 0.27	62.05 ± 0.30	98.70 ± 0.18	87.94 ± 0.12
	SwinUnet	69.91 ± 0.31	53.75 ± 0.45	37.04 ± 0.41	37.28 ± 0.52	98.33 ± 0.16	65.11 ± 0.23
	AAU-net	88.65 ± 0.15	75.48 ± 0.22	60.88 ± 0.26	59.54 ± 0.31	98.08 ± 0.19	87.82 ± 0.13
	TransUnet	87.25 ± 0.17	72.88 ± 0.24	58.05 ± 0.29	59.02 ± 0.33	96.27 ± 0.22	86.55 ± 0.15
	AMSUnet	88.07 ± 0.16	74.36 ± 0.23	59.74 ± 0.28	60.67 ± 0.32	98.25 ± 0.19	87.09 ± 0.14
	Channel Unet	87.68 ± 0.17	74.52 ± 0.24	61.37 ± 0.27	61.81 ± 0.33	**99.39 ± 0.15**	86.40 ± 0.15
	Attention Unet	88.57 ± 0.15	75.15 ± 0.22	60.91 ± 0.26	61.91 ± 0.31	97.79 ± 0.20	87.78 ± 0.13
	UNETR	87.01 ± 0.18	69.01 ± 0.26	53.20 ± 0.32	56.60 ± 0.38	90.17 ± 0.25	86.38 ± 0.16
	UTnet	85.82 ± 0.19	66.19 ± 0.28	49.86 ± 0.35	54.36 ± 0.41	86.59 ± 0.29	85.67 ± 0.18
	HCE-Net (Ours)	**89.22 ± 0.13**	**76.60 ± 0.19**	**62.35 ± 0.24**	**63.09 ± 0.28**	98.55 ± 0.17	**88.42 ± 0.11**
**PCL**	U-Net	98.73 ± 0.10	97.27 ± 0.15	94.88 ± 0.21	96.89 ± 0.24	97.90 ± 0.18	98.98 ± 0.09
	SegNet	98.69 ± 0.11	97.22 ± 0.16	94.62 ± 0.22	97.05 ± 0.23	97.42 ± 0.19	99.07 ± 0.08
	ResUnet	98.80 ± 0.09	97.16 ± 0.15	95.03 ± 0.20	97.76 ± 0.22	97.18 ± 0.17	99.30 ± 0.07
	SwinUnet	98.03 ± 0.15	95.85 ± 0.24	92.13 ± 0.31	95.23 ± 0.28	96.50 ± 0.22	98.50 ± 0.12
	AAU-net	98.75 ± 0.10	97.44 ± 0.15	95.11 ± 0.20	96.96 ± 0.23	98.05 ± 0.17	98.97 ± 0.09
	TransUnet	98.80 ± 0.10	97.37 ± 0.16	93.54 ± 0.25	95.72 ± 0.26	96.13 ± 0.21	99.12 ± 0.08
	AMSUnet	98.49 ± 0.12	97.33 ± 0.16	95.15 ± 0.21	**98.04 ± 0.20**	95.21 ± 0.23	99.33 ± 0.07
	Channel Unet	98.67 ± 0.11	96.95 ± 0.17	94.55 ± 0.22	96.79 ± 0.23	97.66 ± 0.18	98.98 ± 0.09
	Attention Unet	98.10 ± 0.14	94.98 ± 0.25	92.17 ± 0.32	95.84 ± 0.27	95.18 ± 0.24	99.00 ± 0.08
	UNETR	97.89 ± 0.16	96.17 ± 0.23	94.16 ± 0.26	95.15 ± 0.28	94.59 ± 0.26	97.96 ± 0.13
	UTnet	98.01 ± 0.15	97.08 ± 0.17	94.53 ± 0.22	96.83 ± 0.22	**98.62 ± 0.16**	99.00 ± 0.08
	HCE-Net (Ours)	**98.92 ± 0.08**	**97.65 ± 0.13**	**95.49 ± 0.18**	97.84 ± 0.20	97.55 ± 0.16	**99.63 ± 0.06**
**DRIVE**	U-Net	91.23 ± 0.22	65.04 ± 0.34	49.28 ± 0.41	57.47 ± 0.48	77.52 ± 0.39	92.71 ± 0.18
	SegNet	91.76 ± 0.21	59.19 ± 0.39	43.43 ± 0.47	62.10 ± 0.44	59.37 ± 0.45	95.24 ± 0.15
	ResUnet	93.27 ± 0.18	67.38 ± 0.31	51.91 ± 0.38	69.59 ± 0.39	66.67 ± 0.40	96.07 ± 0.13
	SwinUnet	71.86 ± 0.45	29.81 ± 0.62	17.63 ± 0.55	21.68 ± 0.68	57.69 ± 0.49	73.38 ± 0.34
	AAU-net	89.72 ± 0.24	60.58 ± 0.37	44.28 ± 0.44	52.52 ± 0.50	75.58 ± 0.41	91.24 ± 0.19
	TransUnet	86.81 ± 0.28	56.07 ± 0.41	39.89 ± 0.49	45.15 ± 0.55	79.76 ± 0.38	87.56 ± 0.22
	AMSUnet	87.56 ± 0.27	60.48 ± 0.37	44.81 ± 0.44	59.40 ± 0.43	68.83 ± 0.42	89.57 ± 0.20
	Channel Unet	87.60 ± 0.27	61.47 ± 0.36	45.66 ± 0.43	50.74 ± 0.52	83.52 ± 0.35	88.04 ± 0.21
	Attention Unet	87.82 ± 0.26	60.81 ± 0.37	44.98 ± 0.44	50.06 ± 0.53	**81.31 ± 0.37**	88.52 ± 0.21
	UNETR	91.34 ± 0.22	66.30 ± 0.33	50.65 ± 0.40	61.01 ± 0.42	74.45 ± 0.40	93.15 ± 0.17
	UTnet	90.64 ± 0.23	57.44 ± 0.39	41.63 ± 0.47	50.45 ± 0.52	70.03 ± 0.43	92.87 ± 0.17
	HCE-Net (Ours)	**93.40 ± 0.17**	**69.04 ± 0.29**	**53.33 ± 0.36**	**71.43 ± 0.38**	68.58 ± 0.40	**96.14 ± 0.12**

Figs 6 and 7 illustrate the variation curves of the Jaccard coefficient for all comparative models on the TOOTH and DRIVE datasets. The experimental results demonstrate that HCE-Net achieves more stable convergence and delivers superior segmentation performance at the final stage.

**Fig 6:**
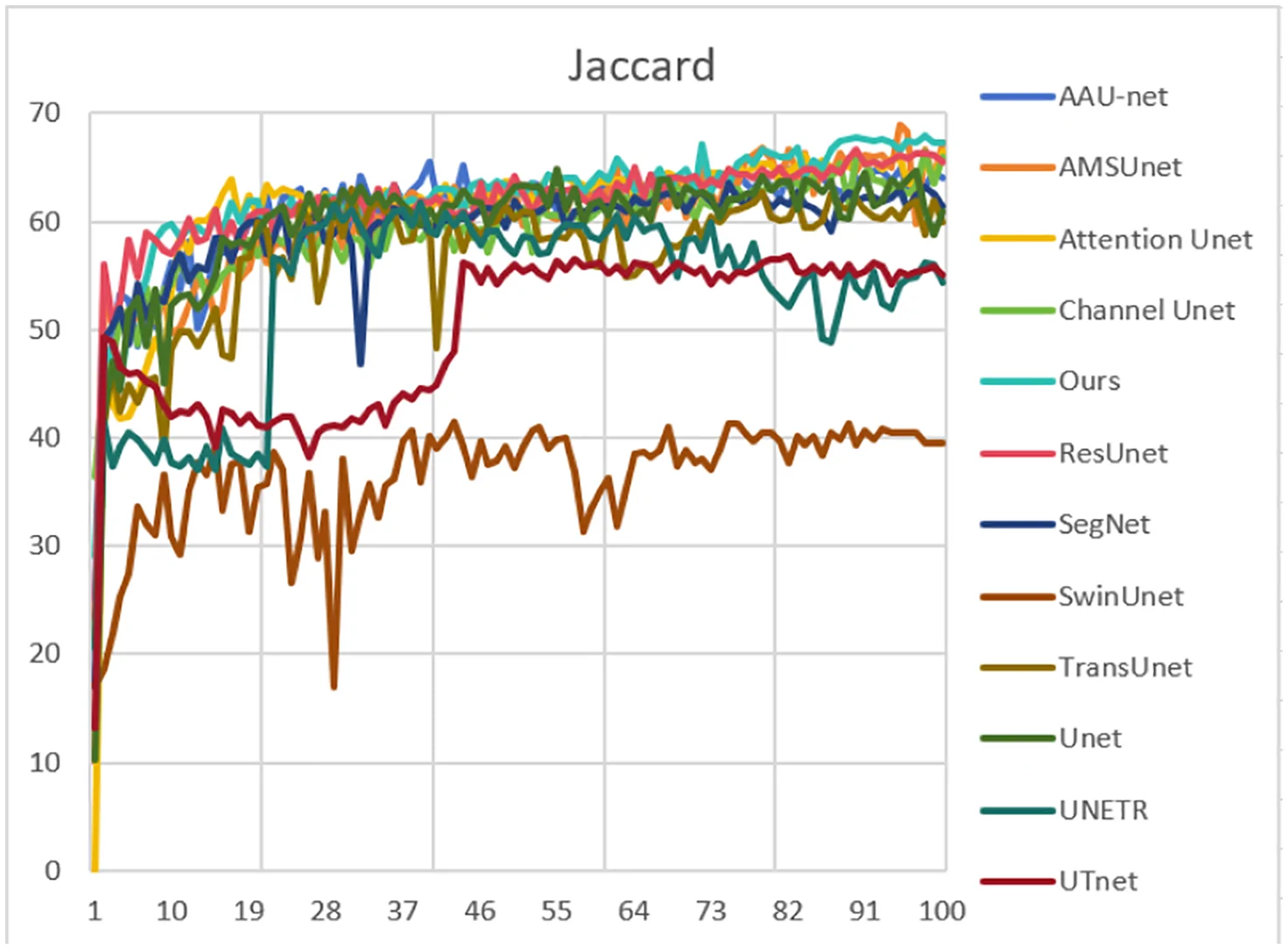
Jaccard Trend Chart on the TOOTH Dataset.

**Fig 7:**
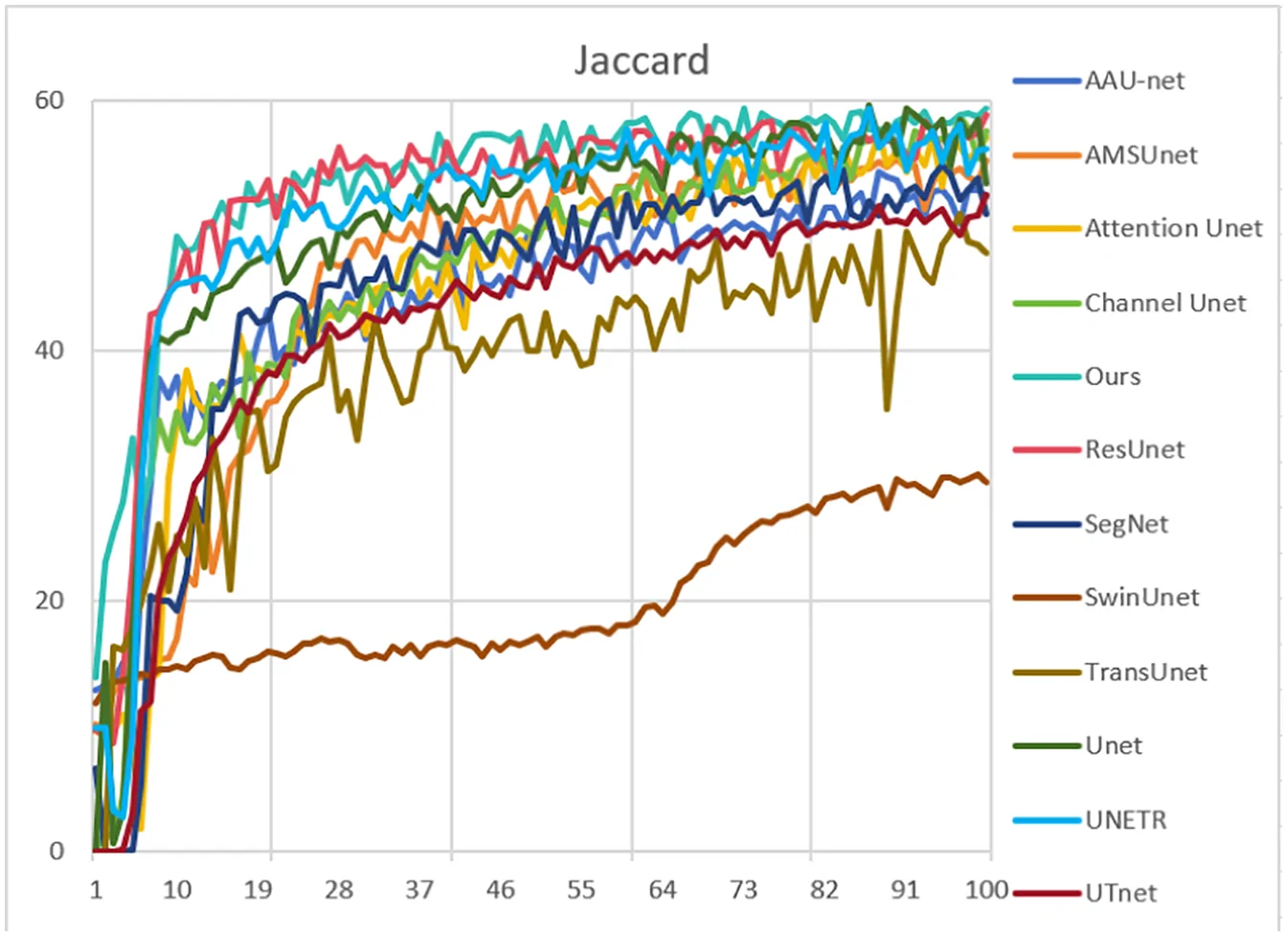
Jaccard Trend Chart on the DRIVE Dataset.

Figs 8, Fig 9 to Fig 10 present the qualitative segmentation comparisons of diverse models across three datasets. On the TOOTH and PCL datasets, HCE-Net yields markedly superior segmentation accuracy for target contours compared with CNN–Transformer hybrid architectures, effectively mitigating edge blurring and over-segmentation artifacts. For the DRIVE dataset, HCE-Net enables more complete extraction of intricate vascular branches and preserves richer critical details than comparative methods. Collectively, quantitative and qualitative evaluations corroborate the comprehensive superiority of HCE-Net over existing CNN-based and CNN–Transformer hybrid models in segmentation precision, fine-detail capture, and overall robustness.

**Fig 8:**
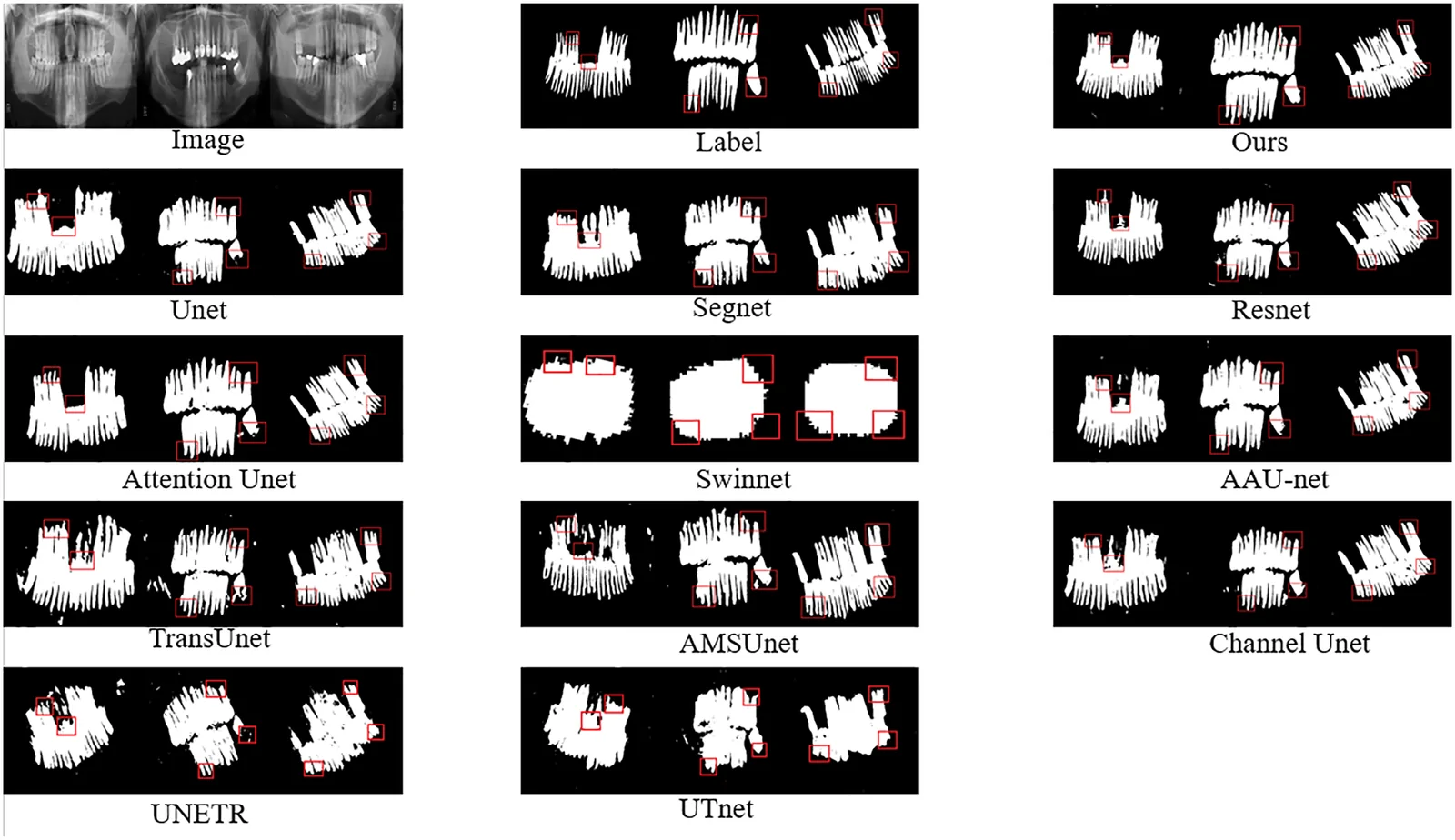
Comparison of Segmentation Effects between HCE-Net and Different Networks on the TOOTH Dataset.

**Fig 9:**
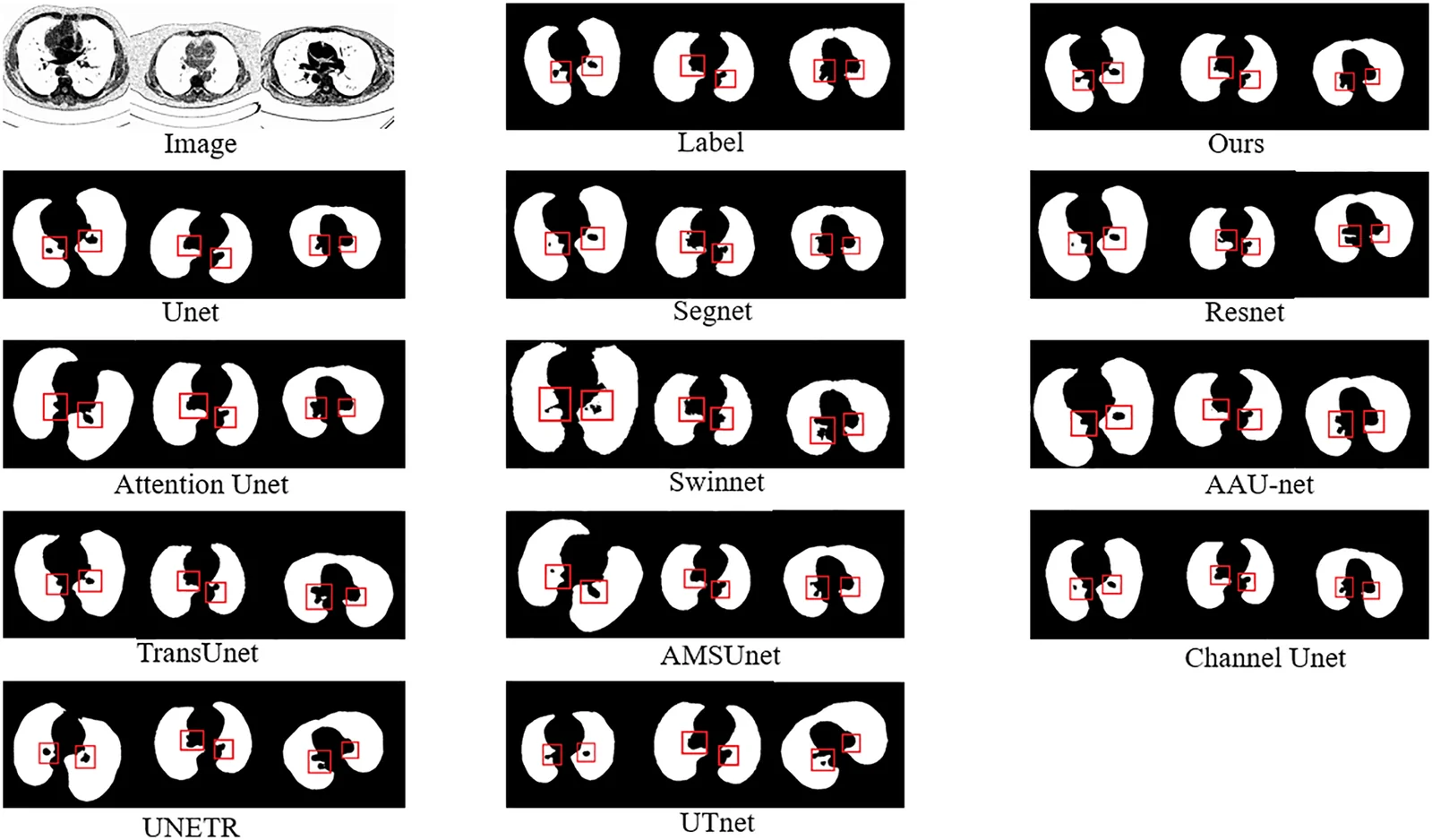
Comparison of Segmentation Effects between MSAE-Net and Different Networks on the PCL Dataset.

**Fig 10:**
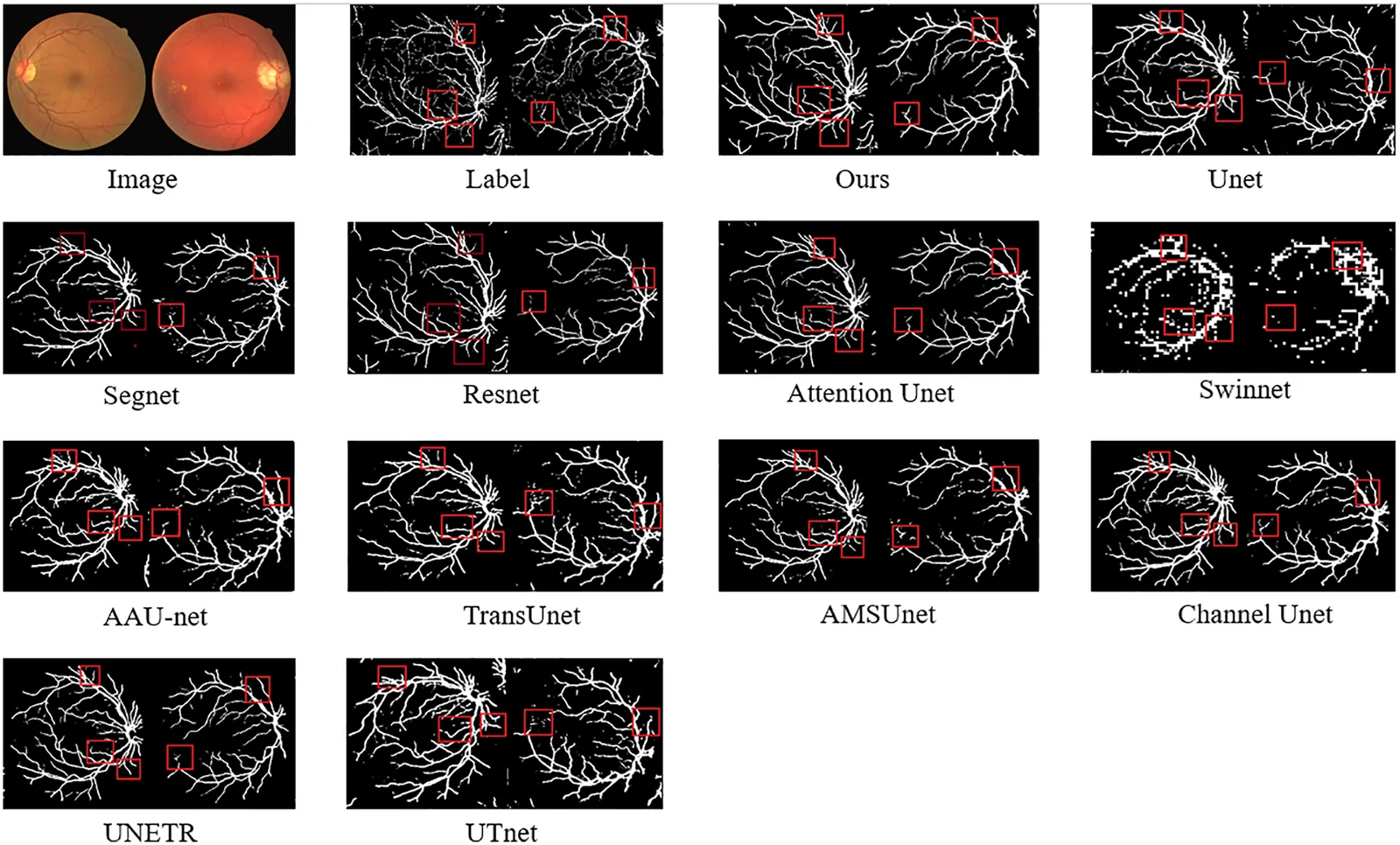
Comparison of Segmentation Effects between MSAE-Net and Different Networks on the DRIVE Dataset.

#### Ablation experiment.

Three groups of ablation experiments are designed in this study to comprehensively validate the efficacy of the proposed Orthogonal Fusion Module, Diversified Receptive Field Module, and the embedded Adaptive Aggregation Connection deployed at corresponding skip connection layers. Controlled variable strategies are adopted for experimental verification to analyze the individual contribution of each component to the overall network performance. The detailed experimental configurations are specified as follows:

(1) OnlyOFM: Only the orthogonal fusion module is retained within the encoder–decoder architecture to validate the functional efficacy of OFM.
(2) OnlyAAC: Merely the adaptive aggregation connection is preserved to verify the performance gains brought by AAC in skip connection optimization.
(3) OnlyDFM: Exclusively the diversified receptive field module is deployed to confirm the capability of DFM in detail retention and segmentation accuracy enhancement.

Table 3 summarizes the quantitative metrics of various network variants derived from ablation experiments on the PCL and DRIVE datasets. Analytical results reveal that each elaborately designed module contributes distinct enhancements to the segmentation capability of the integrated network. Such improvements become particularly pronounced when processing retinal vascular images, which are characterized by intricate edge segmentation and challenging fine‑feature extraction. Notably, the OnlyOFM and OnlyAAC configurations achieve considerable performance gains on the DRIVE dataset, thereby fully corroborating the efficacy and rationality of the proposed orthogonal fusion module as well as the adaptive aggregation connection embedded within skip connections.

**Table 3:** Ablation balance of different networks on PCL and DRIVE datasets.

Datasets	Networks	Acc(%)	Dice(%)	Jaccard(%)	Pre(%)	Recall(%)	Spe(%)
**PCL**	Only OFM	98.78 ± 0.11	97.37 ± 0.16	94.89 ± 0.21	**98.09 ± 0.20**	96.69 ± 0.20	99.42 ± 0.07
	Only AAC	98.89 ± 0.10	97.79 ± 0.15	94.97 ± 0.21	97.14 ± 0.22	96.90 ± 0.19	99.11 ± 0.08
	Only DFM	98.80 ± 0.10	97.53 ± 0.15	95.01 ± 0.20	97.48 ± 0.21	97.25 ± 0.18	99.28 ± 0.07
	U-Net	98.73 ± 0.10	97.27 ± 0.15	94.88 ± 0.21	96.89 ± 0.24	**97.90 ± 0.18**	98.98 ± 0.09
	HCE-Net (Ours)	**98.92 ± 0.08**	**97.65 ± 0.13**	**95.49 ± 0.18**	97.84 ± 0.20	97.55 ± 0.16	**99.63 ± 0.06**
**DRIVE**	Only OFM	92.91 ± 0.19	67.10 ± 0.32	51.43 ± 0.39	70.72 ± 0.40	64.02 ± 0.42	95.13 ± 0.16
	Only AAC	93.03 ± 0.19	67.13 ± 0.32	51.47 ± 0.39	70.62 ± 0.40	63.88 ± 0.42	95.27 ± 0.16
	Only DFM	92.47 ± 0.20	68.77 ± 0.30	50.36 ± 0.40	68.31 ± 0.41	65.70 ± 0.41	94.67 ± 0.17
	U-Net	91.23 ± 0.22	65.04 ± 0.34	49.28 ± 0.41	57.47 ± 0.48	**77.52 ± 0.39**	92.71 ± 0.18
	HCE-Net (Ours)	**93.40 ± 0.17**	**69.04 ± 0.29**	**53.33 ± 0.36**	**71.43 ± 0.38**	68.58 ± 0.40	**96.14 ± 0.12**

Fig 11 visualizes the qualitative segmentation results of different network variants in ablation experiments. Visual observation indicates that the OnlyOFM configuration integrated with the orthogonal fusion module achieves more precise contour delineation and exhibits greater robustness against noise interference. Meanwhile, the OnlyDFM variant equipped with the diversified receptive field module captures abundant contextual features and retains finer anatomical details. Consistent quantitative and qualitative outcomes collectively validate the efficacy of the elaborately designed modules. Furthermore, the optimized reconstruction of skip connections via adaptive aggregation connections substantially strengthens the comprehensive segmentation capacity of the entire model.

**Fig 11:**
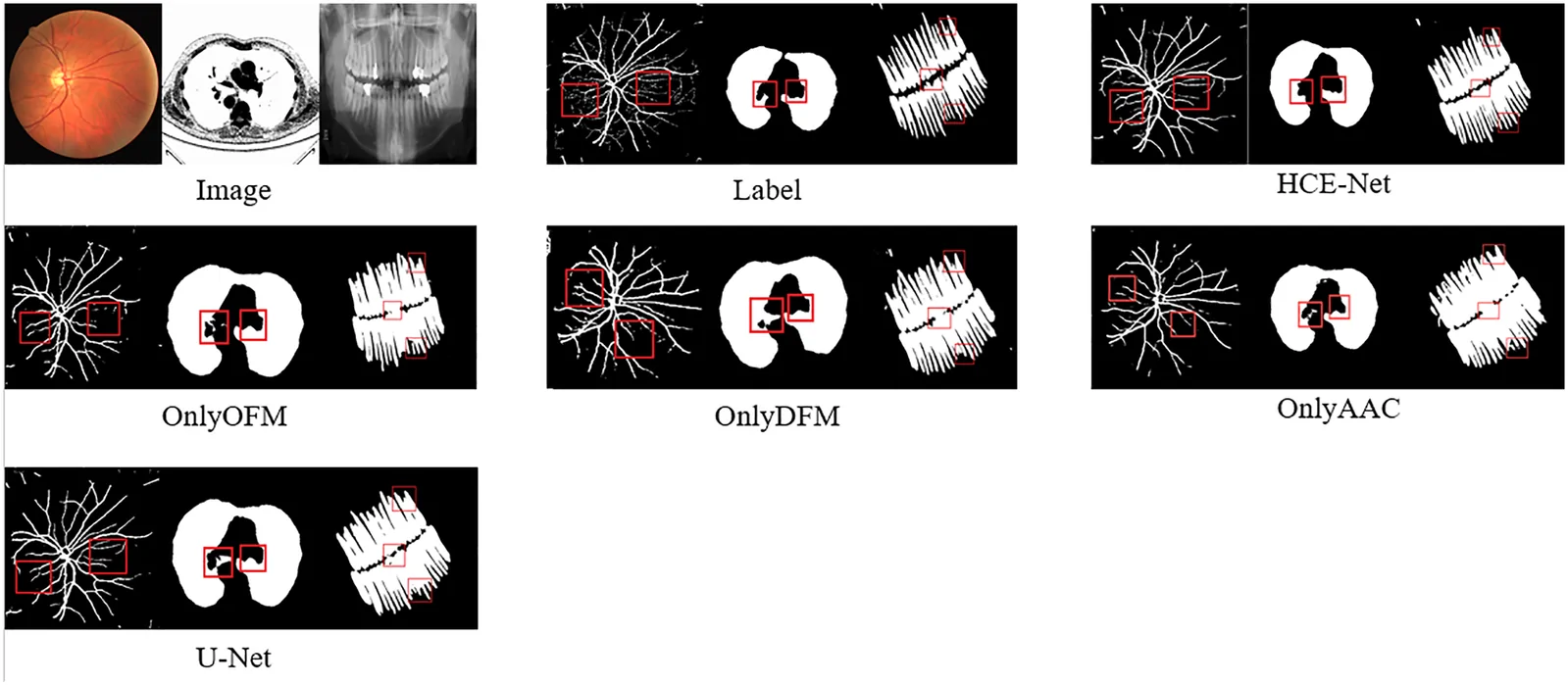
Comparison Chart of Segmentation Effects Among Various Networks in Ablation Experiments.

## Discussion

### Training epochs

Fig 12 presents the loss curves of all comparative models on the TOOTH dataset. As illustrated, the loss of the proposed model tends to stabilize with subtle fluctuations at 100 epochs, indicating satisfactory convergence. Further extension of training iterations would impair computational efficiency and potentially induce performance degradation caused by overfitting. Accordingly, one hundred epochs serve as a rational configuration to balance training sufficiency and computational overhead. Meanwhile, the visual comparison intuitively demonstrates the distinct advantages of our model when addressing datasets with complex edge contours. During the training process, HCE-Net exhibits compelling superiority. The loss declines rapidly within the initial 20 epochs, delivering a convergence rate considerably higher than that of competing models and reflecting efficient training characteristics. At the 100-epoch milestone, our model achieves a prominently lower loss value, which verifies its superior segmentation accuracy. Moreover, the loss curve maintains minimal fluctuation in the late training stage, manifesting stable gradient updates and solidifying the outstanding robustness of HCE-Net.

**Fig 12:**
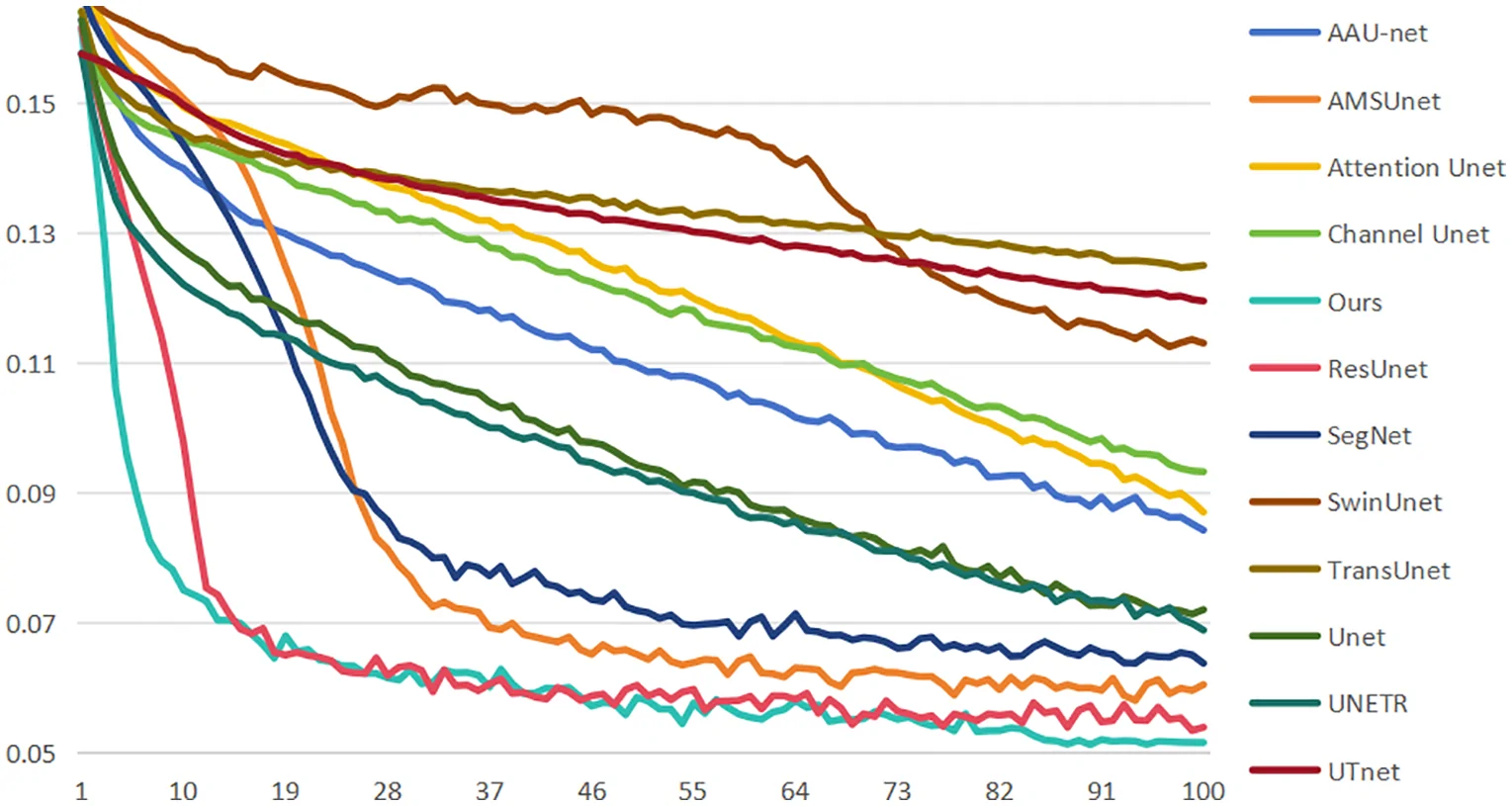
Trend graph of loss functions on the TOOTH dataset.

### Computational complexity and deployment analysis

To verify the practical applicability and scalable deployment potential of HCE-Net, this study quantitatively analyzes its parameter scale, computational complexity (FLOPs), and inference efficiency. Evaluations conducted on an NVIDIA GPU with 24 GB VRAM under the PyTorch framework demonstrate that the model yields a total parameter volume of roughly 18.6M, closely aligned with the original U-Net and free from excessive parameter redundancy. The overall computational burden is limited to 24.5 GFLOPs, ensuring superior operational efficiency. At an input resolution of 256 × 256, the inference latency per single image averages 12 milliseconds, which fully meets the real-time response demands of clinical auxiliary diagnosis. In terms of industrial deployment, HCE-Net is established on standard convolution operators and elaborately designed attention modules, featuring exceptional universal compatibility and convenient practical implementation. The trained model can be exported to ONNX format via PyTorch and further optimized with TensorRT for accelerated inference, enabling stable deployment on clinical workstations, edge computing devices and various mainstream hardware terminals. In essence, HCE-Net achieves substantial improvements in segmentation accuracy while maintaining a compact architecture and high computational efficiency, which endows it with prominent clinical translation value and application prospects.

### Clinical significance and application value

HCE-Net is specifically designed for three typical clinical medical image scenarios—panoramic dental radiographs, lung CT scans, and retinal vascular images—endowing it with clear clinical application value. In panoramic dental images, it enables precise segmentation of dental contours, providing robust support for orthodontic treatment planning and pre-implantation evaluation. In lung CT scans, it accurately extracts the boundaries of the lungs and lesions, serving as a reliable basis for the screening and diagnosis of pulmonary diseases. In retinal vascular images, it effectively segments tiny vascular structures, facilitating the early diagnosis of ocular diseases such as diabetic retinopathy. Benefiting from the enhanced edge contour preservation by the Orthogonal Fusion Module (OFM), the detailed feature retention by the Diversified Receptive Field Module (DFM), and the efficient heterogeneous feature integration by the Adaptive Aggregation Connection (AAC) embedded in skip connections, HCE-Net outperforms traditional models significantly in terms of boundary clarity and detail integrity. It can provide more accurate and stable segmentation results for clinical computer-aided diagnosis systems, demonstrating considerable potential for practical clinical application.

### Future research directions

Our network still has limitations when processing the DRIVE dataset with significant scale differences, as illustrated in Fig 13. In the regions of vascular intersections and complex branches, the extraction of local structural features is limited, and the continuity and integrity of the segmentation results are slightly inferior to those of some comparative networks. This may be attributed to the fixed Sobel kernel adopted in this study and the failure to consider the feature differences across different channels. In the future, we will continue to conduct research and expand the Sobel operator to adapt to different channels.

**Fig 13:**
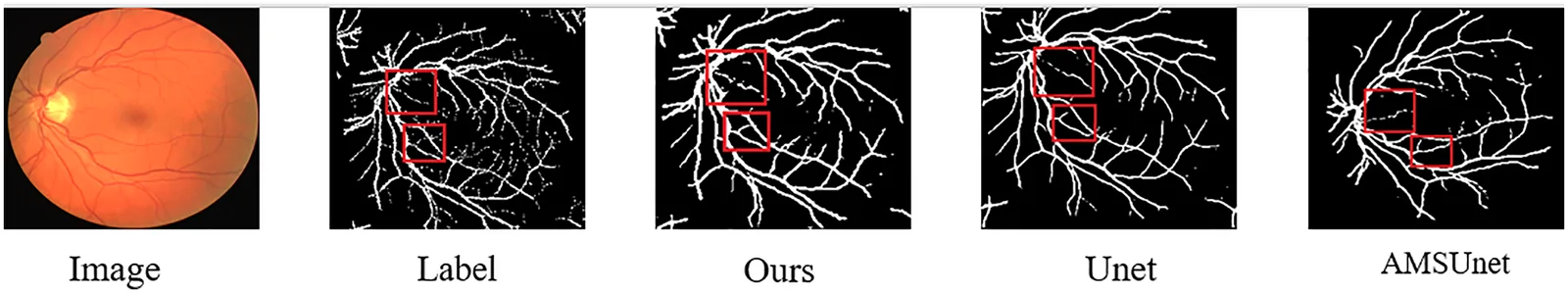
Comparison of results on the DRIVE dataset.

## Conclusion

To address the challenges in medical image segmentation—including the difficulty of a single encoder in balancing edge contour extraction and global detail perception, the ineffective fusion of heterogeneous features, and the deficiencies in boundary precision alongside excessive parameters of CNN-Transformer hybrid models—this study proposes a Heterogeneous Feature Collaborative Extraction Network (HCE-Net) tailored for medical image segmentation. This network adopts a dual-path heterogeneous encoding architecture, decoupling and complementing the extraction of edge contours and global details: the Orthogonal Fusion Module (OFM) integrates Sobel edge priors with multi-scale convolutions to achieve precise delineation of target boundaries; the Diversified Receptive Field Module (DFM) combines channel attention mechanisms with multi-scale convolutions to enhance the retention of texture and contextual information; the Adaptive Aggregation Connection (AAC) is designed to replace traditional linear skip connections, enabling adaptive weighted fusion of two heterogeneous feature streams and effectively resolving issues of cross-path feature conflicts and semantic aliasing. Meanwhile, HCE-Net maintains a lightweight structure and high inference efficiency, endowing it with strong potential for clinical deployment. Comprehensive experimental results verify the feasibility of HCE-Net, fully demonstrating its superior capabilities in edge feature extraction and detail preservation for medical image segmentation. Ablation experiments further validate the effectiveness of each functional module designed within HCE-Net.

### Data and image authorization statement

All datasets used in this study are publicly available.All images adopted in this study are sourced from publicly accessible open datasets and utilized solely for methodological validation and academic research. All visual materials comply with the CC BY 4.0 Creative Commons Attribution License, which permits unrestricted reproduction, distribution, adaptation and commercial application, thereby fully satisfying journal copyright specifications. All data required to reproduce the experimental results are provided. The minimal dataset and code repository can be accessed at https://github.com/qiangxuange/HCE-net/.
