# Supplementary material for: Enhancing medical image segmentation with HCE-Net: A heterogeneous feature collaborative extraction network
Source: PLoS One. 2026 Nov 3;21(11):e0359398. doi: 10.1371/journal.pone.0359398 (PMC13730324; doi:10.1371/journal.pone.0359398)
Supplement: Supplementary file 1 [file pone.0359398.s002.doc]

Response to Editor and Reviewers

Manuscript ID: PONE-D-25-43776Title: Enhancing Medical Image Segmentation with HCE-Net: A Heterogeneous Feature Collaborative Extraction Network

We greatly appreciate the time, effort, and constructive comments provided by the editor and reviewers during the review process. We have carefully addressed all comments and thoroughly revised the manuscript accordingly.

Response to Journal Requirements

**Requirement 1: Please ensure that your manuscript meets PLOS ONE's style requirements, including those for file naming.**

We have revised the entire manuscript in strict accordance with the journal’s formatting requirements, including file naming, heading hierarchy, font formatting, citation standards, and figure and table layout. All formatting issues have been corrected.

**Requirement 2:** **Please remove any funding-related text from the manuscript and let us know how you would like to update your Funding Statement.**

All funding-related statements in the Acknowledgments section have been completely removed.

**Requirement 3: We note that your Data Availability Statement is currently as follows: [All relevant data are within the manuscript and its Supporting Information files.**

We have provided all data necessary to reproduce the experimental results. The data availability statement has been optimized, and the public repository containing the minimal dataset and code is available at: [https://github.com/qiangxuange/HCE-net/](https://github.com/qiangxuange/HCE-net/ )  All datasets used in this study are publicly available, and their official access links have been added in the revised manuscript.

**Requirement 4: When completing the data availability statement of the submission form, you indicated that you will make your data available on acceptance. We strongly recommend all authors decide on a data sharing plan before acceptance, as the process can be lengthy and hold up publication timelines. Please note that, though access restrictions are acceptable now, your entire data will need to be made freely accessible if your manuscript is accepted for publication. This policy applies to all data except where public deposition would breach compliance with the protocol approved by your research ethics board. If you are unable to adhere to our open data policy, please kindly revise your statement to explain your reasoning and we will seek the editor's input on an exemption. Please be assured that, once you have provided your new statement, the assessment of your exemption will not hold up the peer review process**

A complete data sharing plan has been finalized. All raw experimental data, executable code, and model weight files have been uploaded to a public GitHub repository and are freely accessible to all users.

**Requirement 5:** **We note that Figures 8,9,10,11 and 13 in your submission contain copyrighted images. All PLOS content is published under the Creative Commons Attribution License (CC BY 4.0), which means that the manuscript, images, and Supporting Information files will be freely available online, and any third party is permitted to access, download, copy, distribute, and use these materials in any way, even commercially, with proper attribution.**

All experimental images in this study are obtained from public open datasets, including TOOTH, PCL, and DRIVE, which are licensed under the Creative Commons Attribution 4.0 International License (CC BY 4.0). The following statement has been added to the manuscript: All images used in this study are obtained from public datasets and are published under the CC BY 4.0 license. All datasets are fully open and can be used freely for academic research and commercial purposes. No third-party copyrighted images are used in this manuscript, so no additional copyright permission is required.

**Requirement 6: If the reviewer comments include a recommendation to cite specific previously published works, please review and evaluate these publications to determine whether they are relevant and should be cited. There is no requirement to cite these works unless the editor has indicated otherwise.**

We have systematically reviewed the relevant studies recommended by the reviewers, supplemented references from the past three years, and improved the literature review to enhance its comprehensiveness and timeliness.

**Response to Reviewer 1**

**Comment 1: The model concept used is complex. Discuss its complexity, speed, parameters, etc. Analyze its deployment in that context.**

Response: We have added a new independent section 5.2 Computational Complexity and Deployment Analysis to address this comment specifically.

**Comment 2: Why other hybrid CNN-transformer models are not compared? Are they not very similar to the used model concept?**

Response: Based on the original hybrid models including SwinUnet and TransUnet, we have added comparative experiments with other mainstream hybrid models such as UNETR and UTnet. The results confirm that the proposed HCE-Net achieves superior segmentation accuracy over these hybrid architectures.

**Comment 3: Discuss about computational complexity in limitations.**

Relevant discussions on computational complexity have been supplemented in Computational Complexity and Deployment Analysis. Although HCE-Net introduces a multi-branch heterogeneous structure, its overall computational cost remains at the same level as U-Net, demonstrating outstanding lightweight advantages.

**Comment 4: Adaptive Aggregation Connection (AAC) is mentioned in different sections as a new designed mechanism and contribution? Is it not similar to existing concepts?**

Response: We have rewritten this section to clearly define the core innovation of the Adaptive Aggregation Connection module and strengthen the discussion of its novelty and differences.

**Response to Reviewer 2**

**Comment 1: While the proposed HCE-Net introduces a heterogeneous feature extraction mechanism, the manuscript does not clearly explain how the proposed architecture fundamentally differs from existing segmentation models.**

**recent architectures have incorporate feature extraction strategies (multi-stream or hybrid) , including: (U-Net, TransUNet, Swin-UNet). So, the authors should clearly highlight to:**

**• The specific novelty of OFM and DFM**

**• The advantages heterogeneous of feature streams**

Response: We have rewritten the Introduction and Methodology sections to systematically summarize the core innovations of this work.

**Comment 2: The experimental section lacks several important details the following should be provide it such as:**

**• Dataset descriptions**

**• Number of (training, validation, and testing) samples**

**• Procedures of data preprocessing**

**• Image normalization methods**

**• Training strategy**

**• inference speed**

**• Runtime performance**

**Providing these details would allow researchers to replicate the study and improve transparency.**

Response: All missing experimental details have been supplemented in 4.1 and 4.2, including basic dataset information and sample size descriptions, training/validation/test set partitioning rules under five-fold cross-validation, data preprocessing and normalization procedures, training optimization strategies and experimental environment configuration, and model inference speed and efficiency test results. The revised experimental process is complete and reproducible. Relevant discussions on computational complexity have been supplemented Computational Complexity and Deployment Analysis. Although HCE-Net introduces a multi-branch heterogeneous structure, its overall computational cost remains at the same level as U-Net, demonstrating outstanding lightweight advantages.

**Comment 3: The proposed architecture introduces the modules:(OFM, DFM, AAC), the authors should provide a clearer study including how each component improves segmentation performance. For example:**

**- Evaluation Metrics and Statistical Analysis**

**The study reports many evaluation metrics, such accuracy, Dice coefficient, and Jaccard index. But, the statistical reliability is not sufficiently discussed. So, should consider:**

**• Standard deviation values**

**• Confidence intervals**

**• Statistical significance tests**

**These analyses would boosting the validity of the results.**

**- Clinical Relevance and Practical Impact**

**there is limited discussions about practical apps., the researchers should elapesed on: potential clinical cases, computational efficiency, limitations when used in clinical datasets. and this will improve practical relevance of the study.**

Response: All quantitative indicators are uniformly supplemented with mean ± standard deviation. Independent sample t-tests are used to analyze between-group differences with a significance level of p < 0.05. The interpretation of statistical results has been improved to effectively enhance the scientificity and reliability of the experimental data, as stated in the experimental analysis section. The clinical relevance and practical implications are elaborated in Clinical Significance and Application Value.

**Comment 4: There are some sections containe grammatical errors, so need to profecssional editing to improve readability**

Response: The entire manuscript has undergone professional English polishing, and grammatical errors, awkward expressions, and non-standard academic expressions have been systematically corrected.

**Comment 5: Figures need to be more clearer such as: improve labeling, increase resolution and this would help readers to understand the proposed structure.**

Response: All illustrations have been uniformly upgraded to high resolution, with optimized labels, legends, and structural display effects.

**Comment 6: The related work section need to focuse on the last two or three years. and this help to better the proposed work.**

Response: The literature review section has been comprehensively revised, supplemented with the latest research findings, and systematically reviewed the cutting-edge advances of convolutional networks, Transformer architectures, hybrid models, and attention mechanisms in the field of medical image segmentation.

**Summary**

We have addressed all comments from the editor and reviewers point-by-point. We have comprehensively optimized the manuscript and marked all revisions in red in the Revised Manuscript with Track Changes. These revisions have significantly improved the novelty, experimental completeness, data reliability, language standardization, and format compliance of the paper.
