# Supplementary material for: Enhancing medical image segmentation with HCE-Net: A heterogeneous feature collaborative extraction network
Source: PLoS One. 2026 Nov 3;21(11):e0359398. doi: 10.1371/journal.pone.0359398 (PMC13730324; doi:10.1371/journal.pone.0359398)
Supplement: Peer review history

# Peer review: Decision Letter 0

**DOI:** 10.1371/journal.pone.0359398.r001

**Authors:** Pradeep Kumar Gupta

8 Mar 2026

Dear Dr. Wang,

Thank you for submitting your manuscript to PLOS ONE. After careful consideration, we feel that it has merit but does not fully meet PLOS ONE’s publication criteria as it currently stands. Therefore, we invite you to submit a revised version of the manuscript that addresses the points raised during the review process.

Please submit your revised manuscript by Apr 22 2026 11:59PM. If you will need more time than this to complete your revisions, please reply to this message or contact the journal office at plosone@plos.org. When you're ready to submit your revision, log on to https://www.editorialmanager.com/pone/ and select the 'Submissions Needing Revision' folder to locate your manuscript file.

- A letter that responds to each point raised by the academic editor and reviewer(s). You should upload this letter as a separate file labeled 'Response to Reviewers'.
- A marked-up copy of your manuscript that highlights changes made to the original version. You should upload this as a separate file labeled 'Revised Manuscript with Track Changes'.
- An unmarked version of your revised paper without tracked changes. You should upload this as a separate file labeled 'Manuscript'.

If you would like to make changes to your financial disclosure, please include your updated statement in your cover letter. Guidelines for resubmitting your figure files are available below the reviewer comments at the end of this letter.

If applicable, we recommend that you deposit your laboratory protocols in protocols.io to enhance the reproducibility of your results. Protocols.io assigns your protocol its own identifier (DOI) so that it can be cited independently in the future. For instructions see: https://journals.plos.org/plosone/s/submission-guidelines#loc-laboratory-protocols. Additionally, PLOS ONE offers an option for publishing peer-reviewed Lab Protocol articles, which describe protocols hosted on protocols.io. Read more information on sharing protocols at https://plos.org/protocols?utm_medium=editorial-email&utm_source=authorletters&utm_campaign=protocols.

We look forward to receiving your revised manuscript.

Kind regards,

Pradeep Kumar Gupta, Ph.D.

Academic Editor

PLOS One

Journal Requirements:

When submitting your revision, we need you to address these additional requirements.

1. Please ensure that your manuscript meets PLOS ONE's style requirements, including those for file naming. The PLOS ONE style templates can be found at

https://journals.plos.org/plosone/s/file?id=wjVg/PLOSOne_formatting_sample_main_body.pdf and

https://journals.plos.org/plosone/s/file?id=ba62/PLOSOne_formatting_sample_title_authors_affiliations.pdf

2. Thank you for stating the following in the Acknowledgments Section of your manuscript: [This work is supported by the Chengdu University Pattern Recognition and Intelligent Information Processing Sichuan University Key Laboratory open fund (MSSB-2025-15) and Chengdu University Tianfu Culture Digital Innovation Key Laboratory of Sichuan Culture and Tourism Department open project (TFWH-2025-22).]

We note that you have provided funding information that is not currently declared in your Funding Statement. However, funding information should not appear in the Acknowledgments section or other areas of your manuscript. We will only publish funding information present in the Funding Statement section of the online submission form.

Please remove any funding-related text from the manuscript and let us know how you would like to update your Funding Statement. Currently, your Funding Statement reads as follows: [This work was supported by  the Chengdu University Pattern Recognition and Intelligent Information Processing Sichuan University Key Laboratory open fund under Grant MSSB-2025-15 (Recipient: Jiaxi Wang), Chengdu University Tianfu Culture Digital Innovation Key Laboratory of Sichuan Culture and Tourism Department open project under Grant TFWH-2025-22 (Recipient: Jiaxi Wang). The funders had no role in study design, data collection and analysis, decision to publish, or preparation of the manuscript.].

Please include your amended statements within your cover letter; we will change the online submission form on your behalf.

3. We note that your Data Availability Statement is currently as follows: [All relevant data are within the manuscript and its Supporting Information files.]

Please confirm at this time whether or not your submission contains all raw data required to replicate the results of your study. Authors must share the “minimal data set” for their submission. PLOS defines the minimal data set to consist of the data required to replicate all study findings reported in the article, as well as related metadata and methods (https://journals.plos.org/plosone/s/data-availability#loc-minimal-data-set-definition).

For example, authors should submit the following data:

- The values behind the means, standard deviations and other measures reported;

- The values used to build graphs;

- The points extracted from images for analysis.

Authors do not need to submit their entire data set if only a portion of the data was used in the reported study.

If your submission does not contain these data, please either upload them as Supporting Information files or deposit them to a stable, public repository and provide us with the relevant URLs, DOIs, or accession numbers. For a list of recommended repositories, please see https://journals.plos.org/plosone/s/recommended-repositories.

If there are ethical or legal restrictions on sharing a de-identified data set, please explain them in detail (e.g., data contain potentially sensitive information, data are owned by a third-party organization, etc.) and who has imposed them (e.g., an ethics committee). Please also provide contact information for a data access committee, ethics committee, or other institutional body to which data requests may be sent. If data are owned by a third party, please indicate how others may request data access.

4. When completing the data availability statement of the submission form, you indicated that you will make your data available on acceptance. We strongly recommend all authors decide on a data sharing plan before acceptance, as the process can be lengthy and hold up publication timelines. Please note that, though access restrictions are acceptable now, your entire data will need to be made freely accessible if your manuscript is accepted for publication. This policy applies to all data except where public deposition would breach compliance with the protocol approved by your research ethics board. If you are unable to adhere to our open data policy, please kindly revise your statement to explain your reasoning and we will seek the editor's input on an exemption. Please be assured that, once you have provided your new statement, the assessment of your exemption will not hold up the peer review process.

5. We note that Figures 8,9,10,11 and 13 in your submission contain copyrighted images. All PLOS content is published under the Creative Commons Attribution License (CC BY 4.0), which means that the manuscript, images, and Supporting Information files will be freely available online, and any third party is permitted to access, download, copy, distribute, and use these materials in any way, even commercially, with proper attribution. For more information, see our copyright guidelines: http://journals.plos.org/plosone/s/licenses-and-copyright.

We require you to either (1) present written permission from the copyright holder to publish these figures specifically under the CC BY 4.0 license, or (2) remove the figures from your submission:

1. You may seek permission from the original copyright holder of Figures 8,9,10,11 and 13 to publish the content specifically under the CC BY 4.0 license.

We recommend that you contact the original copyright holder with the Content Permission Form (http://journals.plos.org/plosone/s/file?id=7c09/content-permission-form.pdf) and the following text:

“I request permission for the open-access journal PLOS ONE to publish XXX under the Creative Commons Attribution License (CCAL) CC BY 4.0 (http://creativecommons.org/licenses/by/4.0/). Please be aware that this license allows unrestricted use and distribution, even commercially, by third parties. Please reply and provide explicit written permission to publish XXX under a CC BY license and complete the attached form.”

Please upload the completed Content Permission Form or other proof of granted permissions as an "Other" file with your submission.

In the figure caption of the copyrighted figure, please include the following text: “Reprinted from [ref] under a CC BY license, with permission from [name of publisher], original copyright [original copyright year].”

2. If you are unable to obtain permission from the original copyright holder to publish these figures under the CC BY 4.0 license or if the copyright holder’s requirements are incompatible with the CC BY 4.0 license, please either i) remove the figure or ii) supply a replacement figure that complies with the CC BY 4.0 license. Please check copyright information on all replacement figures and update the figure caption with source information. If applicable, please specify in the figure caption text when a figure is similar but not identical to the original image and is therefore for illustrative purposes only.

6. If the reviewer comments include a recommendation to cite specific previously published works, please review and evaluate these publications to determine whether they are relevant and should be cited. There is no requirement to cite these works unless the editor has indicated otherwise.

[Note: HTML markup is below. Please do not edit.]

Reviewers' comments:

Reviewer's Responses to Questions

**Comments to the Author**

1. Is the manuscript technically sound, and do the data support the conclusions?

Reviewer #1: Yes

Reviewer #2: Yes

**********

2. Has the statistical analysis been performed appropriately and rigorously?

Reviewer #1: No

Reviewer #2: I Don't Know

**********

3. Have the authors made all data underlying the findings in their manuscript fully available?

The PLOS Data policy

Reviewer #1: Yes

Reviewer #2: Yes

**********

4. Is the manuscript presented in an intelligible fashion and written in standard English?

Reviewer #1: Yes

Reviewer #2: Yes

**********

Reviewer #1: The paper demonstrates improvements compared to baseline models. the performance is validated don three datasets with extensive evaluation and ablation study. There are following points to address:

1. The model concept used is complex. Discuss its complexity, speed, parameters, etc. Analyze its deployment in that context.

2. Why other hybrid CNN-transformer models are not compared? Are they not very similar to the used model concept?

3. Discuss about computational complexity in limitations.

4. Adaptive Aggregation Connection (AAC) is mentioned in different sections as a new designed mechanism and contribution? Is it not similar to existing concepts?

Reviewer #2: The study proposed method that was evaluated on three datasets, and the results shown that HCE-Net improved segmentation performance compared with many of baseline methods.

The study topic is relevant to the fields of Medical Image Analysis and Deep Learning, the medical image segmentation remains an important problem in clinical decision support systems.

although the study presents an interesting architecture, many issues methodological and presentation must be addressed before the study can be considered for publication.

1- While the proposed HCE-Net introduces a heterogeneous feature extraction mechanism, the manuscript does not clearly explain how the proposed architecture fundamentally differs from existing segmentation models.

recent architectures have incorporate feature extraction strategies (multi-stream or hybrid) , including: (U-Net, TransUNet, Swin-UNet). So, the authors should clearly highlight to:

• The specific novelty of OFM and DFM

• The advantages heterogeneous of feature streams

2- The experimental section lacks several important details the following should be provide it such as:

• Dataset descriptions

• Number of (training, validation, and testing) samples

• Procedures of data preprocessing

• Image normalization methods

• Training strategy

• inference speed

• Runtime performance

Providing these details would allow researchers to replicate the study and improve transparency.

3- The proposed architecture introduces the modules:(OFM, DFM, AAC), the authors should provide a clearer study including how each component improves segmentation performance. For example:

- Evaluation Metrics and Statistical Analysis

The study reports many evaluation metrics, such accuracy, Dice coefficient, and Jaccard index. But, the statistical reliability is not sufficiently discussed. So, should consider:

• Standard deviation values

• Confidence intervals

• Statistical significance tests

These analyses would boosting the validity of the results.

- Clinical Relevance and Practical Impact

there is limited discussions about practical apps., the researchers should elapesed on: potential clinical cases, computational efficiency, limitations when used in clinical datasets. and this will improve practical relevance of the study.

4- There are some sections containe grammatical errors, so need to profecssional editing to improve readability

5- Figures need to be more clearer such as: improve labeling, increase resolution and this would help readers to understand the proposed structure.

6- The related work section need to focuse on the last two or three years. and this help to better the proposed work.

**********

what does this mean?). If published, this will include your full peer review and any attached files.

If you choose “no”, your identity will remain anonymous but your review may still be made public.

**Do you want your identity to be public for this peer review?**  For information about this choice, including consent withdrawal, please see our Privacy Policy

Reviewer #1: No

Reviewer #2: No

**********

[NOTE: If reviewer comments were submitted as an attachment file, they will be attached to this email and accessible via the submission site. Please log into your account, locate the manuscript record, and check for the action link "View Attachments". If this link does not appear, there are no attachment files.]

To ensure your figures meet our technical requirements, please review our figure guidelines: https://journals.plos.org/plosone/s/figures

You may also use PLOS’s free figure tool, NAAS, to help you prepare publication quality figures: https://journals.plos.org/plosone/s/figures#loc-tools-for-figure-preparation.

NAAS will assess whether your figures meet our technical requirements by comparing each figure against our figure specifications.

# Author comment: Author response to Decision Letter 1

**DOI:** 10.1371/journal.pone.0359398.r002

16 Apr 2026

Response to Reviewer 1

Comment 1: The model concept used is complex. Discuss its complexity, speed, parameters, etc. Analyze its deployment in that context.

Response: We have added a new independent section 5.2 Computational Complexity and Deployment Analysis to address this comment specifically.

Comment 2: Why other hybrid CNN-transformer models are not compared? Are they not very similar to the used model concept?

Response: Based on the original hybrid models including SwinUnet and TransUnet, we have added comparative experiments with other mainstream hybrid models such as UNETR and UTnet. The results confirm that the proposed HCE-Net achieves superior segmentation accuracy over these hybrid architectures.

Comment 3: Discuss about computational complexity in limitations.

Relevant discussions on computational complexity have been supplemented in Computational Complexity and Deployment Analysis. Although HCE-Net introduces a multi-branch heterogeneous structure, its overall computational cost remains at the same level as U-Net, demonstrating outstanding lightweight advantages.

Comment 4: Adaptive Aggregation Connection (AAC) is mentioned in different sections as a new designed mechanism and contribution? Is it not similar to existing concepts?

Response: We have rewritten this section to clearly define the core innovation of the Adaptive Aggregation Connection module and strengthen the discussion of its novelty and differences.

Response to Reviewer 2

Comment 1: While the proposed HCE-Net introduces a heterogeneous feature extraction mechanism, the manuscript does not clearly explain how the proposed architecture fundamentally differs from existing segmentation models.

recent architectures have incorporate feature extraction strategies (multi-stream or hybrid) , including: (U-Net, TransUNet, Swin-UNet). So, the authors should clearly highlight to:

• The specific novelty of OFM and DFM

• The advantages heterogeneous of feature streams

Response: We have rewritten the Introduction and Methodology sections to systematically summarize the core innovations of this work.

Comment 2: The experimental section lacks several important details the following should be provide it such as:

• Dataset descriptions

• Number of (training, validation, and testing) samples

• Procedures of data preprocessing

• Image normalization methods

• Training strategy

• inference speed

• Runtime performance

Providing these details would allow researchers to replicate the study and improve transparency.

Response: All missing experimental details have been supplemented in 4.1 and 4.2, including basic dataset information and sample size descriptions, training/validation/test set partitioning rules under five-fold cross-validation, data preprocessing and normalization procedures, training optimization strategies and experimental environment configuration, and model inference speed and efficiency test results. The revised experimental process is complete and reproducible. Relevant discussions on computational complexity have been supplemented Computational Complexity and Deployment Analysis. Although HCE-Net introduces a multi-branch heterogeneous structure, its overall computational cost remains at the same level as U-Net, demonstrating outstanding lightweight advantages.

Comment 3: The proposed architecture introduces the modules:(OFM, DFM, AAC), the authors should provide a clearer study including how each component improves segmentation performance. For example:

- Evaluation Metrics and Statistical Analysis

The study reports many evaluation metrics, such accuracy, Dice coefficient, and Jaccard index. But, the statistical reliability is not sufficiently discussed. So, should consider:

• Standard deviation values

• Confidence intervals

• Statistical significance tests

These analyses would boosting the validity of the results.

- Clinical Relevance and Practical Impact

there is limited discussions about practical apps., the researchers should elapesed on: potential clinical cases, computational efficiency, limitations when used in clinical datasets. and this will improve practical relevance of the study.

Response: All quantitative indicators are uniformly supplemented with mean ± standard deviation. Independent sample t-tests are used to analyze between-group differences with a significance level of p < 0.05. The interpretation of statistical results has been improved to effectively enhance the scientificity and reliability of the experimental data, as stated in the experimental analysis section. The clinical relevance and practical implications are elaborated in Clinical Significance and Application Value.

Comment 4: There are some sections containe grammatical errors, so need to profecssional editing to improve readability

Response: The entire manuscript has undergone professional English polishing, and grammatical errors, awkward expressions, and non-standard academic expressions have been systematically corrected.

Comment 5: Figures need to be more clearer such as: improve labeling, increase resolution and this would help readers to understand the proposed structure.

Response: All illustrations have been uniformly upgraded to high resolution, with optimized labels, legends, and structural display effects.

Comment 6: The related work section need to focuse on the last two or three years. and this help to better the proposed work.

Response: The literature review section has been comprehensively revised, supplemented with the latest research findings, and systematically reviewed the cutting-edge advances of convolutional networks, Transformer architectures, hybrid models, and attention mechanisms in the field of medical image segmentation.

Attachment Submitted filename: Response to Reviewers.doc

# Peer review: Decision Letter 1

**DOI:** 10.1371/journal.pone.0359398.r003

**Authors:** Lorenzo Faggioni

14 Sep 2026

<p>Enhancing Medical Image Segmentation with HCE-Net: A Heterogeneous Feature Collaborative Extraction Network

PONE-D-25-43776R1

Dear Dr. Wang,

We’re pleased to inform you that your manuscript has been judged scientifically suitable for publication and will be formally accepted for publication once it meets all outstanding technical requirements.

Within one week, you’ll receive an e-mail detailing the required amendments. When these have been addressed, you’ll receive a formal acceptance letter and your manuscript will be scheduled for publication.

An invoice will be generated when your article is formally accepted. Please note, if your institution has a publishing partnership with PLOS and your article meets the relevant criteria, all or part of your publication costs will be covered. Please make sure your user information is up-to-date by logging into Editorial Manager at Editorial Manager® and clicking the ‘Update My Information' link at the top of the page. For questions related to billing, please contact billing support.

If your institution or institutions have a press office, please notify them about your upcoming paper to help maximize its impact. If they’ll be preparing press materials, please inform our press team as soon as possible -- no later than 48 hours after receiving the formal acceptance. Your manuscript will remain under strict press embargo until 2 pm Eastern Time on the date of publication. For more information, please contact onepress@plos.org.

Kind regards,

Lorenzo Faggioni, M.D., Ph.D.

Academic Editor

PLOS One

Reviewers' comments:

Reviewer's Responses to Questions

**Comments to the Author**

Reviewer #2: All comments have been addressed

**********

2. Is the manuscript technically sound, and do the data support the conclusions?

Reviewer #2: Yes

**********

3. Has the statistical analysis been performed appropriately and rigorously?

Reviewer #2: Yes

**********

4. Have the authors made all data underlying the findings in their manuscript fully available?

The PLOS Data policy

Reviewer #2: Yes

**********

5. Is the manuscript presented in an intelligible fashion and written in standard English?

Reviewer #2: Yes

**********

Reviewer #2: (No Response)

**********

what does this mean?). If published, this will include your full peer review and any attached files.

If you choose “no”, your identity will remain anonymous but your review may still be made public.

**Do you want your identity to be public for this peer review?**  For information about this choice, including consent withdrawal, please see our Privacy Policy

Reviewer #2: No

**********

# Editor report: Acceptance letter

**DOI:** 10.1371/journal.pone.0359398.r004

**Authors:** Lorenzo Faggioni

PONE-D-25-43776R1

PLOS One

Dear Dr. Wang,

I'm pleased to inform you that your manuscript has been deemed suitable for publication in PLOS One. Congratulations! Your manuscript is now being handed over to our production team.

At this stage, our production department will prepare your paper for publication. This includes ensuring the following:

* All references, tables, and figures are properly cited

* All relevant supporting information is included in the manuscript submission,

* There are no issues that prevent the paper from being properly typeset

You will receive further instructions from the production team, including instructions on how to review your proof when it is ready. Please keep in mind that we are working through a large volume of accepted articles, so please give us a few days to review your paper and let you know the next and final steps.

Lastly, if your institution or institutions have a press office, please let them know about your upcoming paper now to help maximize its impact. If they'll be preparing press materials, please inform our press team within the next 48 hours. Your manuscript will remain under strict press embargo until 2 pm Eastern Time on the date of publication. For more information, please contact onepress@plos.org.

You will receive an invoice from PLOS for your publication fee after your manuscript has reached the completed accept phase. If you receive an email requesting payment before acceptance or for any other service, this may be a phishing scheme. Learn how to identify phishing emails and protect your accounts at https://explore.plos.org/phishing.

If we can help with anything else, please email us at customercare@plos.org.

Thank you for submitting your work to PLOS One and supporting open access.

Kind regards,

PLOS One Editorial Office Staff

on behalf of

Dr. Lorenzo Faggioni

Academic Editor

PLOS One
